# A sumoylation program is essential for maintaining the mitotic fidelity in proliferating mantle cell lymphoma cells

**DOI:** 10.1186/s40164-022-00293-y

**Published:** 2022-07-13

**Authors:** Walter Hanel, Pushpa Lata, Youssef Youssef, Ha Tran, Liudmyla Tsyba, Lalit Sehgal, Bradley W. Blaser, Dennis Huszar, JoBeth Helmig-Mason, Liwen Zhang, Morgan S. Schrock, Matthew K. Summers, Wing Keung Chan, Alexander Prouty, Bethany L. Mundy-Bosse, Selina Chen-Kiang, Alexey V. Danilov, Kami Maddocks, Robert A. Baiocchi, Lapo Alinari

**Affiliations:** 1 Division of Hematology, Department of Medicine, The James Cancer Hospital and Solove Research Institute, The Ohio State University, 460 W 10th Ave, Columbus, OH 43210 USA; 2 Jounce Therapeutics, Cambridge, MA 02139 USA; 3 Proteomics and Mass Spectrometry Facility, The Ohio State University, 460 W. 12th Avenue, Columbus, OH 43210 USA; 4 Department of Radiation Oncology, The James Cancer Hospital and Solove Research Institute, The Ohio State University, 460 W 10th Ave, Columbus, OH 43210 USA; 5 Weil Cornell Medical College, 1300 York Avenue, New York, NY 10065 USA; 6 City of Hope National Medical Center, 1500 E Duarte Rd, Duarte, CA 91010 USA

**Keywords:** UBA2, SAE1, SAE2, UBC9, UBE2I, Topoisomerase, TOP2A

## Abstract

**Background:**

Mantle cell lymphoma (MCL) is a rare, highly heterogeneous type of B-cell non-Hodgkin’s lymphoma. The sumoylation pathway is known to be upregulated in many cancers including lymphoid malignancies. However, little is known about its oncogenic role in MCL.

**Methods:**

Levels of sumoylation enzymes and sumoylated proteins were quantified in MCL cell lines and primary MCL patient samples by scRNA sequencing and immunoblotting. The sumoylation enzyme SAE2 was genetically and pharmacologically targeted with shRNA and TAK-981 (subasumstat). The effects of SAE2 inhibition on MCL proliferation and cell cycle were evaluated using confocal microscopy, live-cell microscopy, and flow cytometry. Immunoprecipitation and orbitrap mass spectrometry were used to identify proteins targeted by sumoylation in MCL cells.

**Results:**

MCL cells have significant upregulation of the sumoylation pathway at the level of the enzymes SAE1 and SAE2 which correlated with poor prognosis and induction of mitosis associated genes. Selective inhibition of SAE2 with TAK-981 results in significant MCL cell death in vitro and in vivo with mitotic dysregulation being an important mechanism of action. We uncovered a sumoylation program in mitotic MCL cells comprised of multiple pathways which could be directly targeted with TAK-981. Centromeric localization of topoisomerase 2A, a gene highly upregulated in SAE1 and SAE2 overexpressing MCL cells, was lost with TAK-981 treatment likely contributing to the mitotic dysregulation seen in MCL cells.

**Conclusions:**

This study not only validates SAE2 as a therapeutic target in MCL but also opens the door to further mechanistic work to uncover how to best use desumoylation therapy to treat MCL and other lymphoid malignancies.

**Supplementary Information:**

The online version contains supplementary material available at 10.1186/s40164-022-00293-y.

## Introduction

Mantle Cell lymphoma (MCL) is a subtype of B-cell non-hodgkin’s lymphoma (NHL) arising from cells of the mantle zone of lymph nodes[1]. The majority of MCL cases exhibit the t(11:14) translocation resulting in overexpression of cyclin D1 as well as mutations within the DNA damage response pathway, most frequently *ATM* or *TP53*, leading to a high degree of genomic instability [2–4]. An elevated proliferative fraction as indicated by Ki-67 positivity by immunohistochemistry of greater than 30% of cells is a poor prognostic factor for patients with MCL with higher relapse rates and shorter overall survival (OS) [5]. Significant advances in novel therapeutics have been made for patients with relapsed/refractory MCL (R/R MCL) including Bruton’s tyrosine kinase (Btk) inhibitors and cellular therapy with anti-CD19 chimeric antigen receptor (CAR) T-cells [6]. However, treatment of patients with R/R MCL remains challenging especially for highly proliferative or Btk inhibitor resistant disease.

Small ubiquitin-like modifier (SUMO) conjugation is a post-translational modification affecting protein–protein interactions, subcellular protein localization, stability, and catalytic activity [7, 8]. After being processed by SUMO proteases (SENPs), SUMO proteins are activated by the heterodimeric SUMO Activating Enzyme (SAE1/2) complex. Following activation, the SAE1/2 complex catalyzes the conjugation of SUMOs onto UBC9 while E3 sumo ligases assist in the transfer of SUMO from UBC9 onto its final protein substrate, thus offering specificity to the pathway. SUMO1 and SUMO2/3 are the most extensively characterized SUMO homologs with each having distinct substrate specificity. SUMO1 proteins are conjugated as monomers while SUMO2/3 proteins may form higher order branching forms via inter-SUMO2/3 conjugation [9].

Aberrant activation of the SUMO pathway occurs across many cancers including several hematologic malignancies, albeit at different levels within the SUMO cascade [10–12]. For example, Burkitt’s lymphoma is characterized by Myc-mediated upregulation of many sumoylation enzymes, including SAE1/2, RanBP2, and PIAS2 with a greater level of total SUMO 2/3 compared to other lymphoma subtypes [13]. In multiple myeloma, UBC9 and PIAS1 are significantly upregulated and associated with poor prognosis [14]. Alternatively, one specific oncoprotein may be sumoylated on a single site which is critical for transformation, such as the case with the PML-RARA fusion protein in acute promyelocytic leukemia [15].

Clinical targeting of sumoylation as an anti-cancer therapy has only recently been achieved with TAK-981 (subasumstat) which is currently being investigated in several ongoing clinical trials as a single agent or in combination with rituximab for patients with relapsed/refractory B-cell NHL (NCT04074330). TAK-981 potently blocks the sumoylation pathway via SAE2-catalyzed formation of a TAK-981-SUMO adduct which binds tightly to a SAE enzyme intermediate state thus potently abolishing further downstream sumoylation conjugation [16]. A recent study has shown TAK-981 to elicit immune activation by way of an immune cell mediated type I interferon response ultimately culminating in lymphoma regression and T-cell memory with immune rejection upon further lymphoma re-challenge [17]. Further recently published studies of TAK-981 demonstrated direct cytotoxic activity in myeloma cells with enhanced sensitivity to dexamethasone through downregulation of miR-551b and miR-25 as well as cell cycle dysregulation and innate immune mediated activity in models of pancreatic cancer [18, 19].

Given the pleiotrophic actions of the sumoylation pathway within different cancer subtypes, we explored the role of the sumoylation pathway within MCL. We found significant upregulation of the sumoylation pathway enzymes SAE1 and SAE2 in MCL primary cells and cell lines. Sumoylation is highly dynamic in proliferating MCL cells but is specifically required at the time of mitosis entry. Importantly, the MCL sumoylation program can be abrogated by TAK-981 resulting in targeting of a broad range of enzymes, most notably DNA topoisomerase 2A, resulting in loss of mitotic fidelity and MCL cell death.

## Methods

### Cell lines, primary samples, drug and cytotoxicity assays

MCL cell lines were grown in standard RPMI-1640 medium supplemented with 10% FBS, and 1% Penicillin–Streptomycin. All cell lines were validated through the University of Arizona Genetic Core using short tandem repeat (STR) validation prior to their use. For B-cell activation experiments, B-cells were isolated from the peripheral blood of healthy donors using negative selection with magnetic beads (Easy Sep Cat#17,954) and cultured in the presence of cytokines [IL-2 (50 ng/mL), IL-4 (10 ng/mL), IL-21 (10 ng/mL) and BAFF (10 ng/mL)] and CD40L expressing fibroblasts (kindly provided by DSMZ) as previously described[20]. For primary MCL samples, PBMCs from patients with relapsed/refractory nodal MCL with leukemic disease were obtained following written informed consent under a protocol approved by the Institutional Review Board of The Ohio State University in accordance with the Declaration of Helsinki. Mononuclear cells were purified by Ficoll-paque and cryopreserved. Cells were thawed and cultured in the presence of CD40L expressing fibroblasts and IL-10 (50 ng/mL), BAFF (50 ng/mL), IGF1 (10 ng/mL), and IL-6 (1 ng/mL) as previously described [21, 22]. Cells were allowed 48 h of recovery after thawing and the percentage of CD19 + , CD5 + cells was confirmed to be 85%. A viability of at least 85% was required before use in downstream assays.

### Cytotoxicity assay

Cells were resuspended by gentle pipetting and 10 uL aliquots were taken and mixed in a 1:1 ratio with Ao/PI stain for cell counting by Nexcelom Bioscience cell counter. For co-culture experiments, cell number was subtracted from a background using irradiated stromal cells with no MCL cell seeding, which typically amounted to less than 5 percent of total cell number. In none of the experiments was loss of stromal cell number or viability observed with TAK-981 (provided by Takeda Development Center Americas, Inc., Lexington MA) treatment.

### Cell cycle analysis

Cells were fixed in 70% ethanol overnight at − 20 degrees. Cells were washed 3 times and stained with Propidium iodide (Sigma) in 0.1% Triton X-100 supplemented with RNase for 1 h and analyzed by FACS. Cells were gated first for FSC vs SSC followed by FSC-A vs FSC-H to gate out doublets. Cell cycle phases were quantified from PI histograms using Kaluza version 2.1.

### Gene knock-down using shRNA

Lentiviral particles were produced by transient transfection of Lenti-X 293 T cells (Takara Bio USA Inc.) with 15 μg of vector DNA in a pLKO backbone (TRCN0000272902; TRCN0000007472, Mission SAE2 shRNA, Sigma) along with the packaging constructs psPAX (15 μg), and pVSG (3 μg) using Lipofectamine 2000 (Thermo Fisher Scientific). Virus containing supernatants were collected at 24 h after transfection and used to infect MCL cell lines using spinoculation by mixing cells with the indicated lentiviral particles and centrifuging at 3900 rpm at 32 °C for 90 min. After 1 day, cells were then selected for 48 h in puromycin (1ug/mL). Cells were then washed and allowed 3 days for recovery prior to downstream assays. For SAE1 knockdown, after lentiviral transduction from viruses obtained from transfection of SAE1 human shRNA plasmids (Origene, Cat#TL315567), cells were GFP sorted 1 day after transfection and downstream assays were carried out after three days from sorting.

### Live cell microscopy, confocal microscopy, and Proximity Ligation Assays (PLA)

Please see Additional file 1: Methods.

### scRNA sequencing

To allow for an intra-patient expression comparison of non-malignant cells to lymphoma cells, thawed MCL primary samples were washed with PBS and CD19 negative cells were enriched by collection of flow through after a CD19 positive selection using Easy Sep Magnetic particles (Stem Cell Technologies, Cat.17854). Cells were fixed in methanol and stored at − 80C before further processing as a single batch. Refer to Additional file 1: Methods for further library preparation, sequencing, data processing and analysis.

### Immunoblot, immunoprecipitation, and Orbitrap MS

Cells were lysed in RIPA buffer (10 mM Tris–HCl [pH 7.4], 150 mM NaCl, 1% Triton X-100, 0.1% SDS and 1% sodium deoxycholate) containing protease and phosphatase inhibitors (all from Sigma), 20 mM of N-ethylmalemide, and 100 mM of iodoacetamide. Cell lysates were clarified by centrifugation. Western blots were captured by either using an enhanced chemiluminescence substrate for detection of scant HRP (Thermo Fisher Scientific) or by LI-COR Biosciences Odyssey Infrared Imaging System using IRDye antibodies (Two-color 181 multiplex detection). For further details on antibodies, immunoprecipitation and Orbitrap MS, see Additional file 1: Methods.

### Animal studies

NSG mice were purchased from Jackson. TAK-981 powder was suspended in sterile H2O with 20% 2-hydroxypropyl-beta-cyclodextriin (HPBC) and administered at 7.5 mg/kg twice weekly via tail vein. For derivation of a novel MCL patient derived xenograft (PDX), 10^7^ of cryopreserved cells from pt #2 were injected into NSG mice. Upon mice meeting end removal criteria (ERC), human MCL cells were recovered from mouse spleens. Cryopreserved PDX cells from passage 6 were used for the TAK-981 treatments in this study. Treatment (vehicle (n = 5) or TAK-981 7.5 mg/kg intravenously twice weekly (n = 10) was initiated either 14 days (Jeko-1) or 28 days (PDX) post engraftment and resumed until ERC was met. All animal studies were approved by the OSU Institutional Animal Care and Use Committee.

### Statistical analysis

Results for viability, cell cycle phases, and microscopy quantification are presented as mean ± standard error of the mean (SEM). Unless otherwise stated in the figure legend, significance was determined using two sample t test (two tailed) with a significance level of p = 0.05 with at least three independent experiments. For mouse survival studies, data is displayed as Kaplan–Meier curves with significance determined by log rank with a significance level of p = 0.05. For statistical analyses of scRNA-seq and mass spectrometry, refer to the specific subsections describing these techniques. For in vivo studies, mouse numbers for each group (n = 5 vehicle, n = 10 TAK-981) were chosen to provide 80% power to detect a median difference in extension of survival of 7 days (standard deviation of 5 days within each group).

## Results

### Sumoylation is significantly dysregulated in MCL

**Fig. 1:**
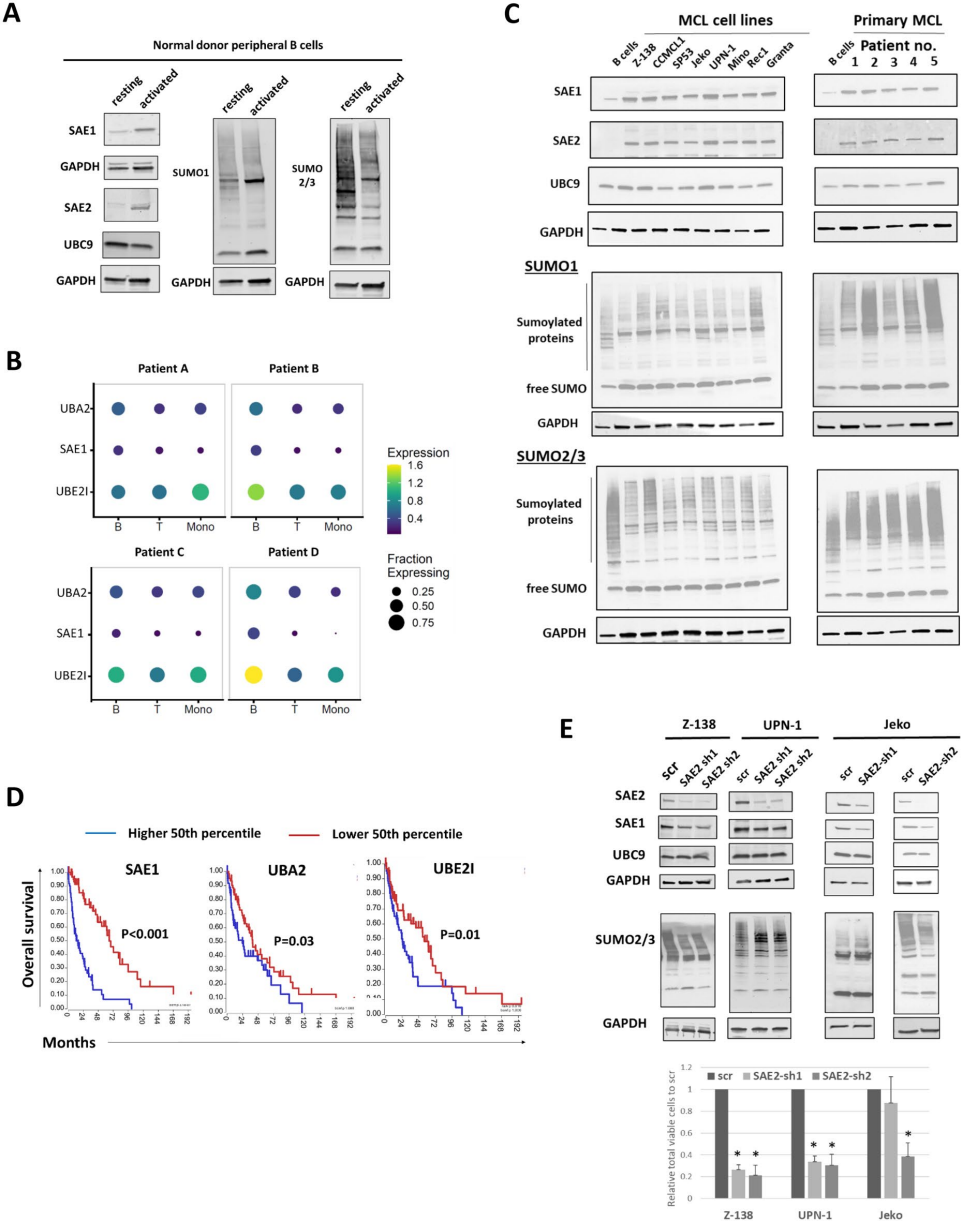
Sumoylation is significantly upregulated in MCL cells and is essential for survival. **A** B-cells were isolated from normal peripheral blood and activated for 3 days (See Methods). Lysates were prepared and blotted for the indicated proteins. **B** Dot plots of the relative expression levels of UBA2 (SAE2), SAE1, and UBE2I (UBC9) within different cell subtypes from peripheral blood samples from four patients with MCL with leukemic disease. **C** MCL cell lines (left, n = 8) and primary MCL cells isolated from the peripheral blood of patients with leukemic disease (right, n = 5) and normal donor resting B-cells were blotted for the indicated proteins. **D** Survival curves of MCL patients (n = 102) stratified by the upper and lower 50^th^ percentile of expression of SAE1, SAE2, and UBC9 using the R2 Genomic analysis and visualization platform. **E** Z-138 (left) UPN-1 cells (middle) or Jeko cells (right) were transduced with lentiviruses containing short hairpins targeting SAE2. After 2 days of puromycin selection, lysates were prepared and blotted for SAE1, SAE2, UBC9, SUMO1 or SUMO2. Three days after removal from selection, total viable cells were quantified. (n = 3 independent experiments for each, * p < .05)

The sumoylation enzymes SAE1/2 and free SUMO proteins are highly upregulated during stages of mouse proliferation particularly during times of B-cell activation including the pre-B cell stage and in the germinal center [13]. To confirm this in human cells, we activated B-cells isolated from the peripheral blood of normal donors using cytokine stimulation and a CD40L expressing stromal cells as previously described [20]. B-cell activation was confirmed with CD80, CD86, and HLA-DR, cell cycle entry with PI staining, as well as increase in p-Btk and c-myc levels (Additional file 1: Fig. S1). We then assessed the central enzymatic components of the sumoylation pathway, including SAE1/2, UBC9 as well as their SUMO1 and SUMO2/3 profiles (Fig. 1A). We found significant induction of SAE1 and SAE2 in activated proliferating B-cells compared to resting B-cells, with changes in several bands within the sumoylation profiles of both SUMO1 and SUMO2/3, with overall more pronounced changes in the SUMO2/3 profile (Fig. 1A, right). Given the central importance of B-cell activation in MCL through the BCR pathway [23], we hypothesized that MCL cells may also significantly recruit SAE1 and SAE2 to drive their proliferation and survival and thus may be a novel therapeutic vulnerability in proliferative MCL. We performed scRNA sequencing on the peripheral blood from 4 patients with relapsed/refractory nodal MCL in leukemic phase. As expected, UMAP clustering showed individual cell subsets of T-cell, NK cells, monocytes, and B cells as indicated by their marker expression (Additional file 1: Figures S2, S3). Subclustering of patient B-cells showed one large cluster and a second much smaller cluster likely representing malignant and normal B-cells, respectively, given the high expression of CCND1 in the malignant cluster and similar UMAP coordinates of the smaller cluster compared to reference B-cells (n = 8) (Additional file 1: Fig. S2, S3). We examined the expression of the sumoylation enzyme genes *SAE1*, *UBA2* (SAE2) and *UBE2I* (UBC9) in individual immune cell subsets and malignant B-cells. We found near ubiquitous expression of *UBE2I* across cell subtypes without any consistent difference among patient samples (Fig. 1B). In contrast, malignant B-cells had consistently higher overall cell numbers with *SAE1* and *UBA2* expression as well as higher expression levels of *SAE1* and *UBA2* compared to normal immune cell subsets. We next looked at the sumoylation enzymes at the protein level. Compared to normal resting B cells, we found higher levels of SAE1 and SAE2 in MCL cell lines and primary MCL samples, while UBC9 levels were similar, consistent with the pattern found in the scRNA sequencing data and that of activated B-cells (Fig. 1C, top). When comparing the sumoylation profiles of malignant and normal resting B-cells, there were significant shifts in the profile of the SUMO1 and SUMO2/3 sumoylated proteins with enhanced levels of many higher molecular weight sumoylated proteins. Particularly striking was the SUMO1 profile of primary MCL cells with an abundance of SUMO1 modified proteins. Using publicly available gene expression data with associated patient outcomes [24], we found that MCL patients (n = 122) with higher expression of either *SAE1*, *UBA2*, or *UBE2I* expression had a worse overall survival, with *SAE1* expression levels showing the greatest separation of survival curves, further demonstrating the relevance of sumoylation pathway in MCL (Fig. 1D). Genetic knock-down of the catalytic E1 subunit, SAE2, in the MCL cell lines Jeko, Z-138 and UPN-1 by shRNA led to a significant loss in the total number of viable cells, thus supporting the importance of sumoylation in promoting MCL cell survival (Fig. 1E) Similarly, downregulation of SAE1 in Jeko cells also had a similar reduction of total viable cells (Additional file 1: Fig. S4). Taken together, these findings show that the sumoylation pathway is highly active in MCL, particularly at the level of SAE1 and SAE2, and may serve as a therapeutic target in this disease.

### TAK-981 leads to desumoylation and MCL cell death in vitro and in vivo

**Fig. 2:**
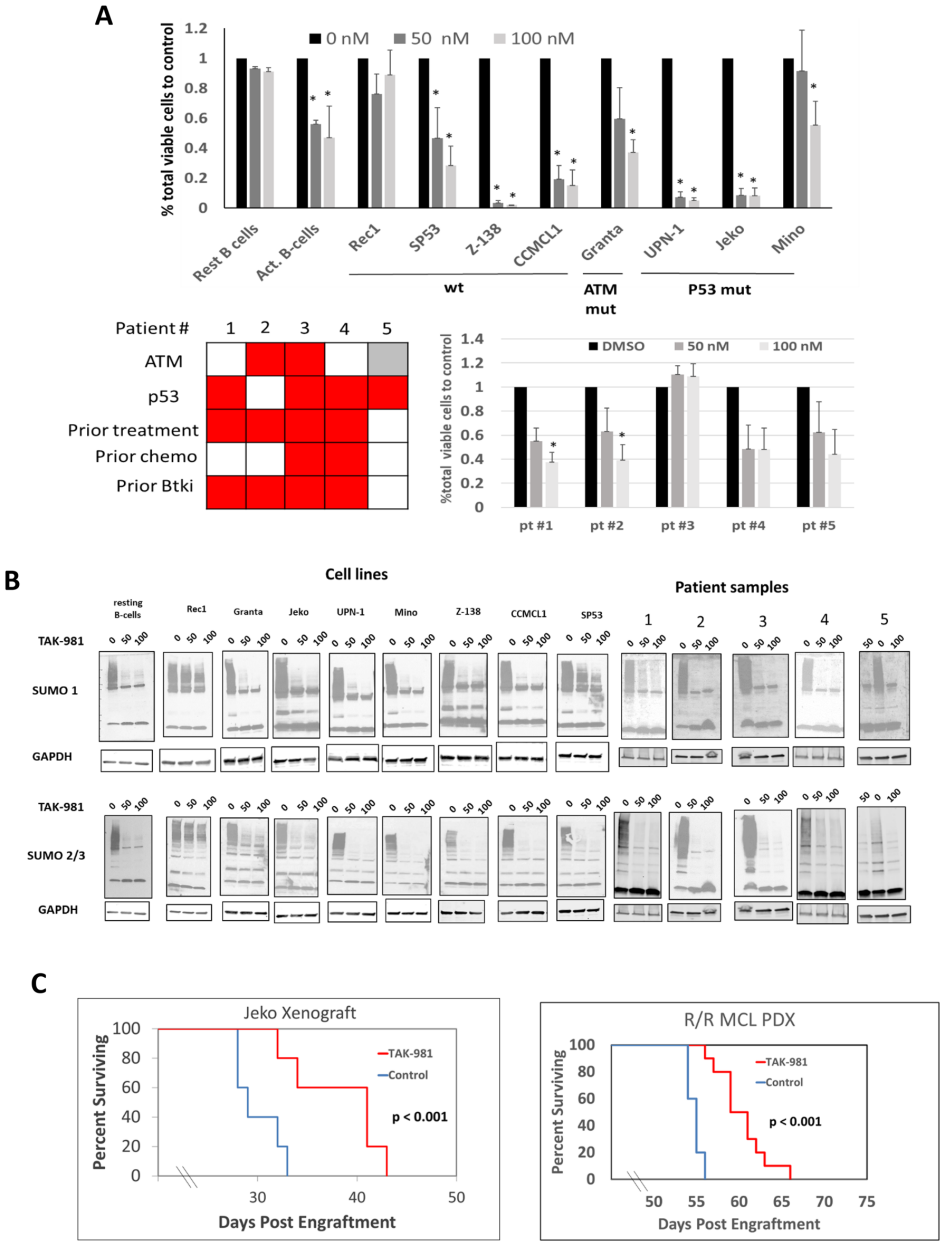
The sumoylation inhibitor TAK-981 leads to loss of sumoylation and cell death in MCL cell lines and patient samples **A** Normal donor resting and activated B-cells, MCL cell lines (top) and primary MCL samples (bottom) were treated with DMSO, 50, and 100 nM of TAK-981. Percent of total viable cells relative to DMSO treatment was determined. (n = 3 independent experiments for each, * p < .05). Bottom left, For each patient sample, the presence of TP53 or ATM mutation or the indicated clinical characteristic is shaded in red. Gray indicates incomplete data. **B** MCL cell lines were treated with DMSO, 50 nM or 100 nM of TAK-981 for 24 h. Cell lysates were blotted for total sumoylated proteins with an anti-SUMO1 (top) or anti-SUMO2/3 (bottom) antibody. C. NSG mice were engrafted with 1 × 10^7^ of either Jeko cells (left) or MCL PDX cells (right) and treated starting on day 14 (Jeko) or day 28 (PDX) post engraftment with either vehicle (n = 5) or TAK-981 7.5 mg/kg intravenously twice weekly (n = 10). Kaplan–Meier analysis shows that TAK-981 treatment yielded a statistically significant increase in survival compared to controls in both models (p < 0.001)

Given the pronounced upregulation of SAE2 and sumoylated proteins in MCL and their necessity for MCL cell survival, we next wanted to know if targeting SAE2 with a clinical grade small molecule inhibitor would lead to anti-tumor activity in MCL. TAK-981 is a potent inhibitor of the sumoylation pathway which forms covalent adducts with SUMO proteins, a reaction that is directly catalyzed by SAE2 [16]. Accumulation of SUMO-adducts subsequently abrogates all downstream sumoylation by competitive inhibition of SAE1/2. We treated a panel of MCL cell lines, primary MCL patient samples, and normal donor peripheral blood resting and activated B-cells with TAK-981 (50 and 100 nM, 3 days) and determined effects on cell viability (Fig. 2A). We found a significant loss in total viable cells in 7 of 8 cells lines relative to DMSO control with a highly significant reduction in 4 cell lines (CCMCL1, Z-138, Jeko, and UPN-1 with < 20% total viable cells with 50 nM TAK-981) and a moderate level of activity in 3 MCL cell lines (SP53, Granta, Mino- < 50% total viable cells with 100 nM of TAK-981) and activated B-cells (Fig. 2A). No significant loss of viability was seen in either normal B-cells or Rec1 cells. Similar to the MCL cell lines, treatment with TAK-981 resulted in significant cell death in 4 of 5 primary MCL patient samples (Fig. 2A bottom), including 3 of 4 ibrutinib-resistant MCL patients. Of note, TAK-981 sensitivity was not dependent on having an intact *TP53* or *ATM* pathway in both MCL cell lines and patient samples (Fig. 2A). We found potent loss of sumoylation with TAK-981 treatment in 7 of 8 MCL cell lines and 5 of 5 primary MCL patient samples (Fig. 2B). The only exception was Rec1 cells which demonstrated only a minor loss of SUMO conjugation, thus explaining the lack of efficacy of TAK-981 in this cell line. Using an antibody that recognizes TAK-981-SUMO-adducts (MIL 113–67-2), we found SUMO adduct formation in all cell lines and normal B-cells (Additional file 1: Fig. S5).

We further extended our in vitro findings in two separate MCL xenograft models. In the first experiment, Jeko cells were engrafted by tail vein injection into NSG mice (n = 5 per group) and randomized to receive either vehicle control or TAK-981 treatment (7.5 mg/kg via tail vein injection, twice weekly, beginning 14 days post engraftment). As shown in Fig. 2C (left panel), treatment with TAK-981 resulted in a statistically significant extension in median OS compared to vehicle control animals (29 vs 41 days, p < 0.001). Also, in a newly established relapsed/refractory MCL PDX model generated in our lab from a patient with acquired ibrutinib resistance (See Methods), TAK-981 treatment (n = 5 vehicle, n = 10 TAK-981, 7.5 mg/kg twice weekly via tail vein injection, beginning 28 days post engraftment) resulted in a statistically significant extension in median OS (55 vs 61 days, p < 0.001) (Fig. 2C, right). These results demonstrate activity of TAK-981 in preclinical models of MCL.

### Loss of SUMOylation leads to mitotic dysregulation in MCL

**Fig. 3:**
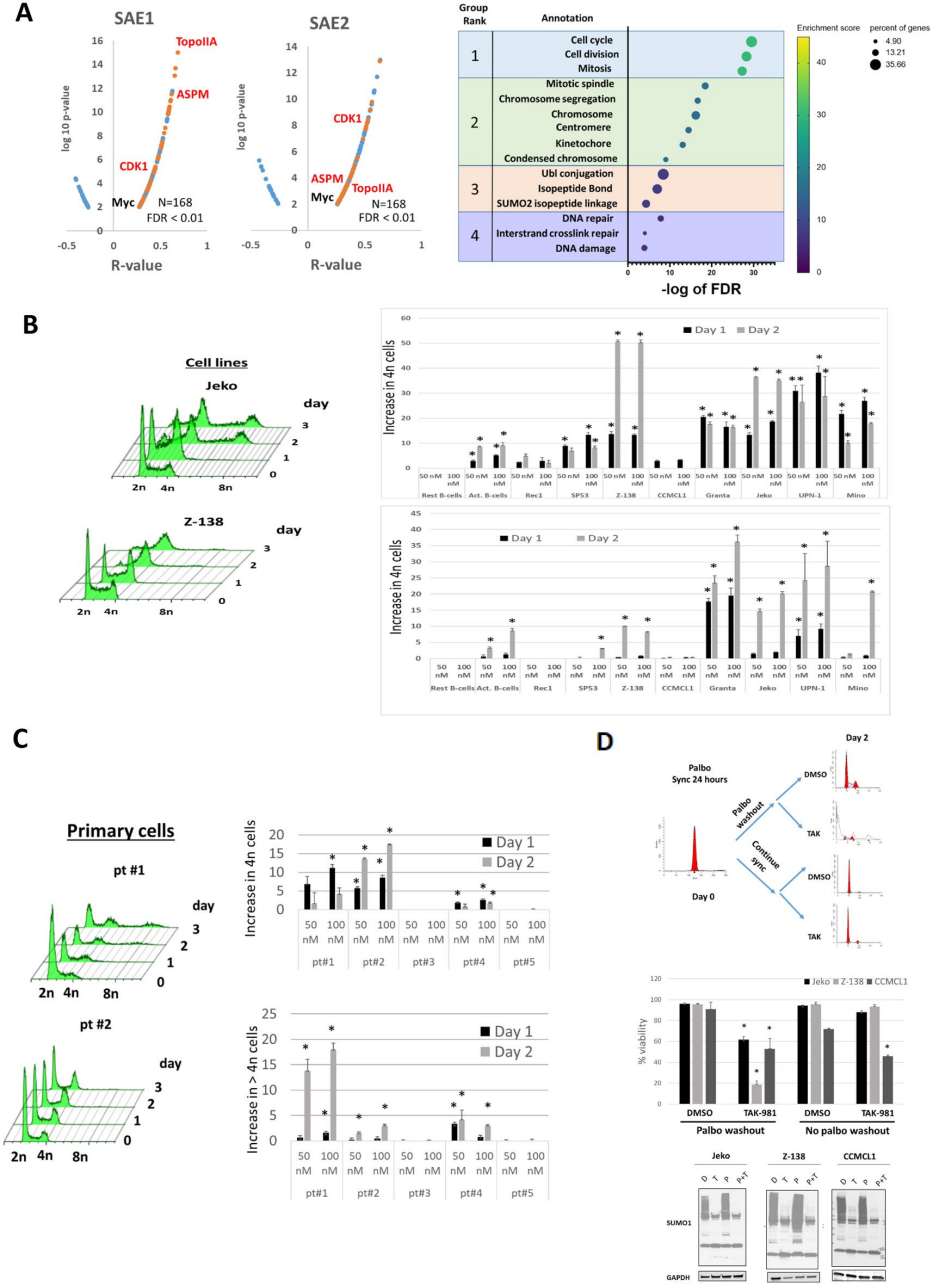
Loss of sumoylation results in mitotic dysregulation with accumulation of polyploid cells **A** (left). Volcano plot of genes with a statistically significant association with SAE1 and SAE2 (FDR < 0.01). (right) DAVID analysis was performed on the statistically significant gene list. The top gene groups and enrichment scores are shown. **B **(right). Normal and activated B-cells and MCL cell lines were treated with DMSO, 50 nM or 100 nM of TAK-981 and cells were collected daily. Cells were fixed, stained with PI and cell cycle analysis was performed. Representative DNA profiles are shown for the indicated cell lines and primary samples treated with 100 nM of TAK-981. Quantification of 2n cells relative to DMSO control (top, right) at 24 h and percentage polyploid cells (bottom, right) after 48 h of TAK-981 treatment at the indicated concentrations (n = 3 independent experiments per cell line). **C** Primary MCL samples were treated and analyzed as in **B** (n = 3 independent experiments per sample). **D** (Left) The indicated cell lines were treated with palbociclib (200 nM) for 24 h and then released into DMSO, palbociclib, TAK-981 or palbociclib + TAK-981. (right top) Percentage viable cells was quantified after 72 h from the time of release from the initial palbociclib treatment (n = 3 independent experiments for each cell line, * p < 0.05). (right bottom) Western blot confirming equivalent losses of sumoylated proteins with TAK-981 in the presence or absence of TAK-981

To gain mechanistic insight into the role of the sumoylation pathway in MCL, we looked for functional groups at the level of transcription that significantly correlated with both SAE1 and SAE2 expression using publicly available expression data and the R2 genomic analysis and visualization platform[24]. We found a total of 168 significantly correlated genes (FDR < 0.01), 146 positively correlated and 22 negatively correlated (Fig. 3A, left). Using database for annotation visualization and integrated discovery (DAVID) analysis of this gene set, we found a significant enrichment of genes involved in cell cycle (cluster 1, 36% of genes), with enrichment in genes involved in chromosome segregation and centromeric functions (cluster 2, 20% of genes) (Fig. 3A, right). Three genes (*TOP2A*, *CDK1*, and *ASPM*) previously discovered to be part of an MCL proliferation gene signature [24] positively correlated with *SAE1* and *SAE2* expression, with *TOP2A* being the most highly significantly correlated gene within the *SAE1* set with a r = 0.65, log_10_ p = 13.0 (Fig. 3A, left).

Given these results, we assessed whether mitotic dysregulation was an important contributor to the efficacy of TAK-981 treatment in MCL. We evaluated DNA profiles over time in MCL cell lines and primary MCL patient samples treated with TAK-981 (Fig. 3B, C). After 24 h of treatment with TAK-981, a significant accumulation of cells with a 4n DNA content occurred in 6 of the 8 cell lines tested (Fig. 3B, top), with a smaller accumulation of 4n activated B-cells and no change in resting B-cells. There was also rapid accumulation of cells with higher ploidy numbers (> 4n) in 5 of the 8 cell lines (Fig. 3B, bottom). CCMCL1 (TAK-981 sensitive) and Rec1 (TAK-981 resistant) were the only two cell lines with no significant changes in their DNA profile with TAK-981 treatment. A similar pattern was observed in primary MCL patient samples that were undergoing proliferation (pt#1, 2, 4, Fig. 3C, Additional file 1: Fig. S6). Of the 2 primary MCL samples that were not actively proliferating (pt #3 and pt#5), one was resistant to TAK-981 (pt#3) while the other was sensitive (p#5) (Fig. 2A).

Given that in some MCL cases like CCMCL and pt#5, we found a cell cycle independent drop in viability upon loss of sumoylation, we wanted to assess the contribution of cell cycle dependent effects in MCL cases with clear mitotic dysregulation. To this end, we used Jeko and Z-138 cells (deregulated cell cycle), versus CCMCL cells (serving as a negative control given lack of cell cycle deregulation). We synchronized cells in the G1 phase of the cell cycle with palbociclib, a CDK4/6 inhibitor [25] for 24 h, followed by either cell cycle entry (palbociclib washout) or continued G1 arrest (no washout) each in the presence of either TAK-981 or DMSO (Fig. 3D). As expected, we found significant loss of viability in both Z-138 and Jeko cells with release from palbociclib to TAK-981, similar to the effect of treatment in unsynchronized cells. However, this loss of viability was prevented by maintaining cells in G1 state (no palbociclib washout), despite significant loss of sumoylation (Fig. 3D, bottom right). In contrast, CCMCL1 cells were not protected when held in G1, likely due a cell cycle-independent effect of TAK-981 in these cells. Overall, these results show that while MCL response to TAK-981 is pleiotrophic, cell cycle dysregulation appears to be a predominant mechanism of cell death upon loss of sumoylation.

### MCL cells with inhibited sumoylation retain a normal G2M kinase cascade but undergo asymmetric division and cell death during mitosis

**Fig. 4:**
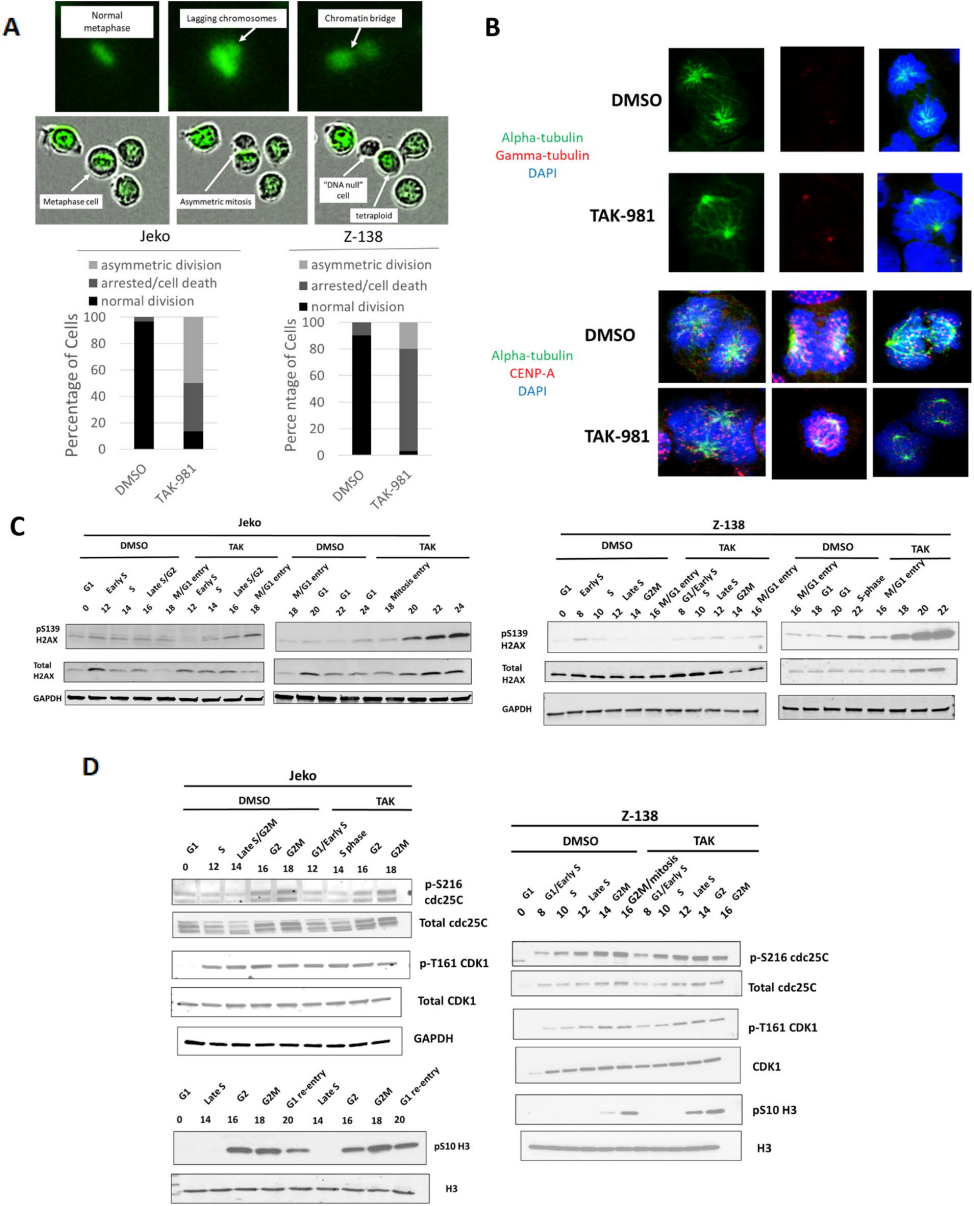
Loss of sumoylation results in severe mitotic dysfunction with significant DNA damage upon mitosis entry. **A** Jeko cells or Z-138 cells (bottom) were transduced with a H2B-GFP construct. Cells were synchronized with palbociclib (500 nM, 24 h), washed and released into DMSO or TAK-981 (100 nM). GFP and phase-contrast images were acquired every 5 min over 24 h. (Top) Representative series of images of Jeko cells transitioning through mitosis in the presence of TAK-981. Jeko (bottom left) or Z-138 (bottom right) were followed and the result of mitosis for individual cells was quantified (n = 50 per group, two independent experiments for each). **B** Representative confocal microscopy images of mitotic Jeko cells (top) for alpha tubulin (green), gamma-tubulin (red), and DAPI (blue) and mitotic Jeko (bottom left, middle) and Z-138 cells (bottom right) for alpha tubulin (green), CENP-A (red), and DAPI (blue). **C**, **D** Jeko (left) and Z-138 cells (right) were synchronized with palbociclib (500 nM) and treated with either DMSO or TAK-981 (100 nM). Lysates were prepared at the indicated time points after washout. The corresponding phase of the cell cycle based on the DNA profiles obtained from fixed, PI stained cells from each of the time points is shown (see Additional file 1: Figures S7, S8). Lysates were blotted for the indicated proteins

The mechanism of mitotic dysregulation upon loss of sumoylation has varied depending on the cell type being examined, with phenotypes ranging from a complete block in G2M to mitosis delay [26, 27], with no detailed analysis of mitotic dysregulation as of yet in lymphoma. To understand the specific mechanism of mitotic dysregulation in MCL following treatment with TAK-981, we incorporated a GFP-tagged H2B by lentiviral transduction into Jeko and Z-138 cells and followed cells by live cell microscopy to monitor progress through mitosis and cytokinesis (Fig. 4A). Each cell line was synchronized with palbociclib followed by washout and release into DMSO or TAK-981 with concurrent monitoring of the DNA profiles. We found nearly equivalent numbers of cells arriving at G2 with TAK-981 treatment as compared to DMSO (Additional file 1: Figures S7, S8) with no evidence of S phase delay. We found evidence of aberrant mitoses indicated by chromatin bridge formation and congression failure as evidenced by chromosomes outside of the metaphase plate following treatment with TAK-981 in both cell lines (Fig. 4A, top). While DMSO-treated Jeko cells underwent normal symmetric division, a large fraction of Jeko cells treated with TAK-981 underwent asymmetric cell division, many times with one cell showing absence of H2B-GFP fluorescence and the other showing persistent fluorescent signal (Fig. 4A, bottom). For Z-138 cells, although initial prophase figures could be identified, progression to metaphase plates and cytokinesis was not as frequent as in Jeko cells, suggesting many cells underwent cell death earlier in mitosis, explaining the lower rate of polyploidy cells compared to Jeko treated cells (Fig. 3A). To further complement our live cell microscopy findings, we assessed the integrity of the centrosomes, microtubule, and centromeres using confocal microscopy (Fig. 4B). Interestingly, both DMSO and TAK-981-treated Jeko and Z-138 cells showed the presence of bipolar centrosomes with spindle formation. However, there was near absence of anaphase figures following treatment with TAK-981. Consistent with live cell microscopy results, we found knob and bleb formation at the nuclear periphery with many cells showing centromeres still present at the periphery despite having bipolar spindle formation, further suggesting significant metaphase congression deficits (Fig. 4B).

Next, we performed a cell cycle time course analysis of DNA damage (pS139 H2AX, Fig. 4C) as well as several key phosphorylated proteins during the G2M transition in synchronized MCL cell lines (Fig. 4D). We annotated the cell cycle phase for each time point by indicating the cell cycle phase with the largest percentage of cells at that particular time point (Additional file 1: Figures S7, S8). Both Jeko and Z-138 cells had rapid accumulation of p-H2AX and total H2AX upon transitioning into mitosis compared to DMSO treated cells without a significant amount of p-H2AX present during S phase. Z-138 cells had a more pronounced increase in total and p-H2AX upon mitosis entry compared to Jeko cells, consistent with their higher rate of mitotic cell death compared to Jeko cells (Fig. 4C). We found that induction of total and phosphorylated levels of the G2M transition proteins CDC25C (pS216), CDK1 (pT161), and H3 (pS10) remained intact despite TAK-981 treatment in both Jeko and Z-138 cells (Fig. 4D) with an overall higher level of pH3S10 in TAK-981 treated MCL cells likely due to a delay of cells through mitotic progression. These results suggest that loss of sumoylation in MCL cells results in significant DNA damage and cell death upon entry into mitosis with relatively little DNA damage during S phase.

### SUMO conjugation is necessary prior to but not during mitosis for proper MCL mitotic division

**Fig. 5:**
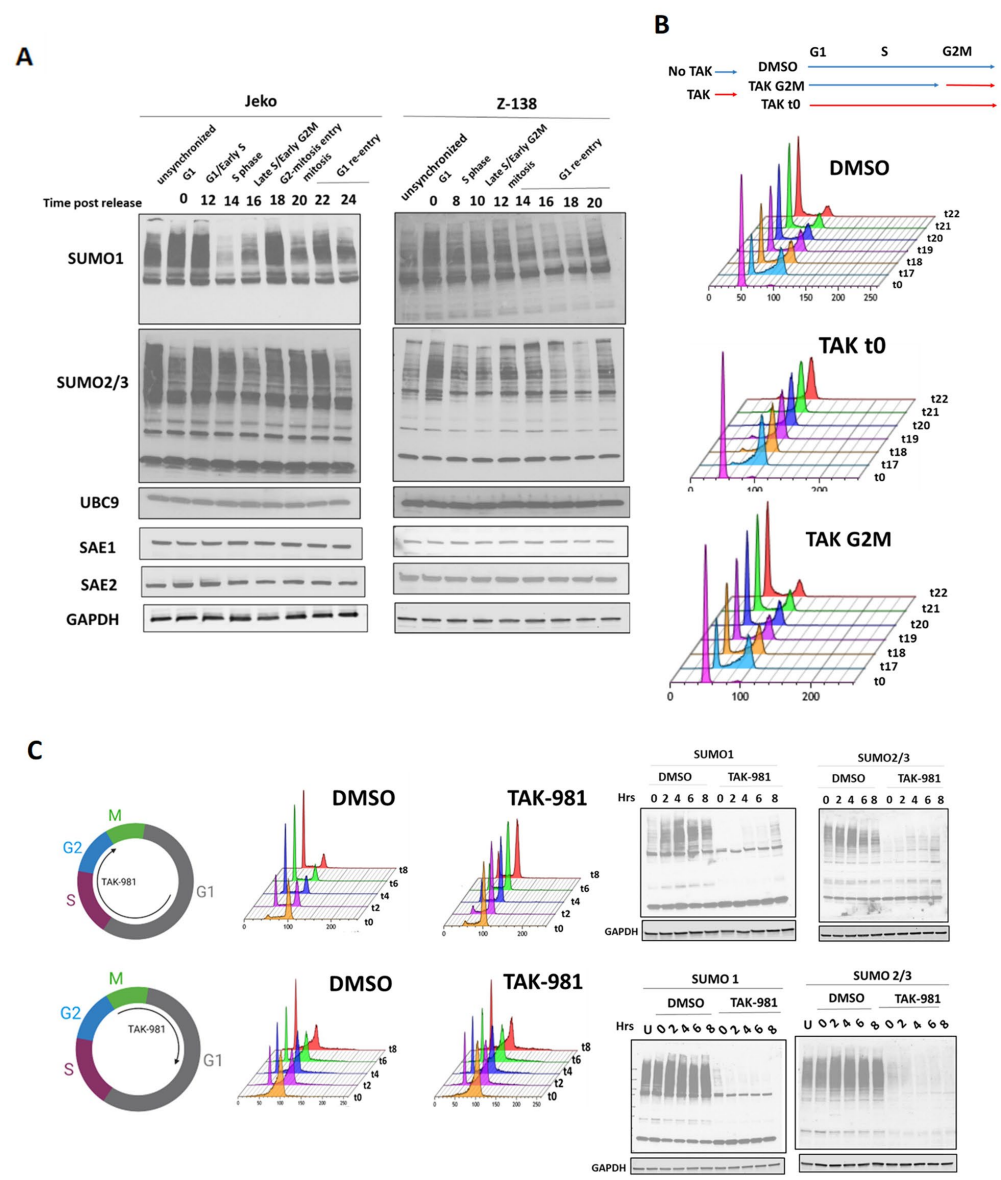
Inhibition of SUMO conjugation during S phase is required for proper mitotic division in MCL cells **A** Jeko (left) or Z-138 (right) cells were synchronized with palbociclib (500 nM) and treated with either DMSO or TAK-981 (100 nM) and lysates were prepared at the indicated time points after washout. The corresponding phase of the cell cycle based on the DNA profiles obtained from fixed, PI stained cells from each of the time points is shown. Lysates were blotted for SUMO1, SUMO2/3, SAE1, SAE2, UBC9, and GAPDH (loading control). **B** Jeko cells were synchronized with Palbociclib (500 nM) for 24 h and washed from drug. Cells were either treated with DMSO or TAK-981 immediately after Palbociclib washout (t = 0) or prior to the start of G2M (17 h) (Top) Schematic showing the time of addition of TAK-981 for each experimental condition. (Bottom) DNA profiles as a function of time. **C **(Top) Jeko cell were synchronized with Palbociclib (500 nM, 24 h) followed by drug washout and treatment with nocodazole (50 ng/mL) either in the presence of DMSO or TAK-981 (100 nM) for 24 h. Both drugs were then washed out and cells were collected every 2 h for cell cycle analysis and for protein for SUMOylation levels. (Bottom) Jeko cell were synchronized with Palbociclib (500 nM, 24 h) followed by drug washout and treatment with nocodazole (50 ng/mL) for 24 h. Cells were then treated with either DMSO or TAK-981 for 3 h. Cells were then washed from both drugs and treated with either DMSO or TAK-981 and cells were collected every 2 h for cell cycle analysis and for protein for SUMOylation levels

To identify potential sumoylated proteins important for mitosis in MCL cells, we first evaluated the levels of total sumoylated proteins at individual stages during progression of the cell cycle in Jeko and Z-138 cells (Fig. 5A). SUMO1 levels in both cell lines were high in G1 and decreased upon transition into S phase which was followed by an increase in levels during G2M. Compared to SUMO1 conjugation, SUMO2/3 conjugated proteins generally showed less cell cycle fluctuation but did have a modest increase in both cell lines occurring slightly after the increase in SUMO1 conjugation. Most notably, there was loss of many SUMO1 and SUMO2/3 substrates upon exit from mitosis into G1, suggesting a possible mitotic sumoylation program in MCL cells. Levels of SAE1, SAE2, and UBC9 remained relatively stable throughout the cell cycle, suggesting a significant amount of cell cycle regulation occurring at the level of the SUMO conjugated substrates themselves.

Given these dynamic changes, we further defined the timing of the necessity for sumoylation for cell cycle progression by adding TAK-981 at the time of G2M entry in Jeko and Z-138 cells, thereby bypassing TAK-981 treatment during S phase (Fig. 5B). We found that addition of TAK-981 after S phase did not result in accumulation of cells with a 4n DNA content in both MCL cell lines, in contrast to when TAK-981 was added upon entry into S phase. To exclude the possibility that the former condition did not allow adequate time for desumoylation, we used a different approach in which Jeko and Z-138 cells were synchronized initially with palbociclib followed by release into nocodazole thus blocking their entry into mitosis. When TAK-981 was added at the time of palbociclib washout (G1 to prometaphase), as expected, we found an increase in 4n cells upon washout from nocodazole (Fig. 5C top, and Additional file 1: Fig. S9 top). However, if TAK-981 was added after 24 h of nocodazole treatment followed by washout, (Fig. 5C, bottom and Additional file 1: Fig. S7 bottom), we found that cells exited into G1 with 2n DNA content at a similar rate as in DMSO treated cells. To ensure adequate loss of sumoylation levels under in this experimental setup, MCL cells were maintained in the presence of TAK-981 for 3 h prior to nocodazole washout, with loss of sumoylation confirmed by immunoblotting (Fig. 5C, right, Additional file 1: Fig. S9, right). Taken together, these findings indicate that the activation of a sumoylation program targeting proteins involved in chromosome segregation occurs prior to mitosis entry and that abrogation of SUMO conjugation prior to mitosis entry is a necessary prerequisite for mitotic dysregulation to occur.

### MCL cells display a diverse multifunctional SUMOylation program upon mitosis entry which is lost with TAK-981 treatment

**Fig. 6:**
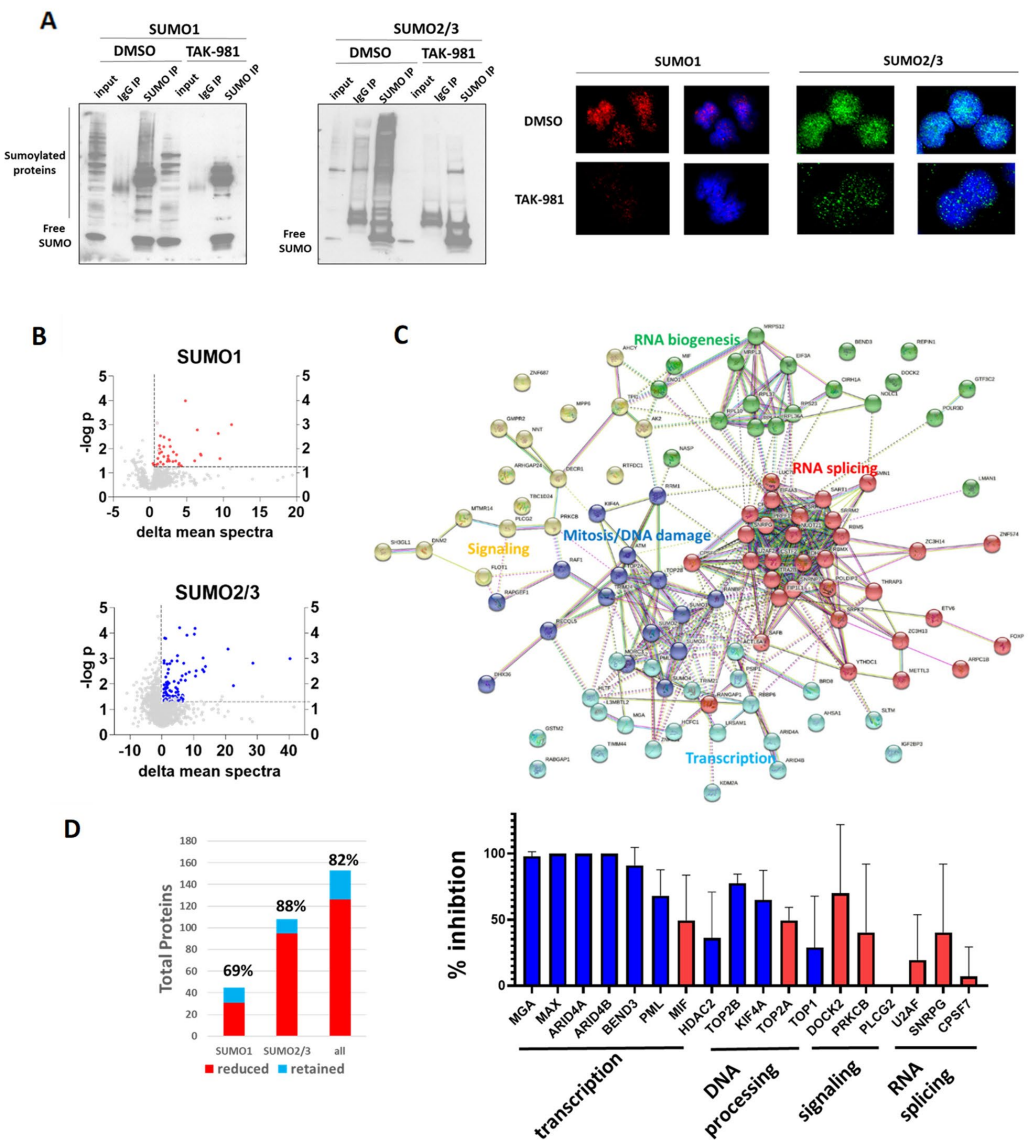
MCL cells turn on a sumoylation program prior to mitosis entry which can be targeted by TAK-981. **A** Jeko cell were synchronized with Palbociclib (500 nM, 24 h) followed by drug washout and treatment with nocodazole (50 ng/mL) either in the presence of DMSO or TAK-981 (100 nM) for 24 h. (left) Lysates were immunoprecipitated with antibodies directed towards SUMO1, SUMO2/3 or respective IgG controls. (right) Microscopy was performed for SUMO1 or SUMO 2/3. **B** (top left). Immunoprecipitants from each condition in **A** were analyzed by Orbitrap MS (see “Methods” section) and proteins identified as being significantly enriched (n = 3 independent experiments, p = 0.05) in SUMO IP compared to IgG are shown (left, SUMO1 = red; SUMO2/3 = blue). **C** Protein–protein interaction network of all sumoylation enriched proteins created by STRING using k-means clustering. **D** Average percent inhibition (n = 3) of sumoylated proteins with TAK-981 (100 nM) based on Orbitrap MS spectral data of individual proteins identified in B (SUMO1 substrates = red; SUMO2/3 substrates = blue)

Previous studies have identified numerous sumoylated proteins involved in mitotic programs [28, 29], however a mitotic sumoylation program has not been evaluated in lymphoma to date. We wanted to not only identify the sumoylation program in MCL cells upon mitosis entry but also discover which proteins within this program were lost upon TAK-981 treatment. To enrich for these proteins, we first synchronized Jeko in G1 with palbociclib and released cells into either DMSO or TAK-981 and held these cells just prior to mitosis entry with nocodazole (see Fig. 5C, top). Immunoprecipitation performed with SUMO1, SUMO2/3, and IgG controls with and without TAK-981 (Fig. 6A, left) showed significant enrichment of proteins that were readily lost with TAK-981 treatment, confirming the specificity of the immunoprecipitation. Microscopy for either SUMO1 or SUMO2/3 showed a predominantly nuclear pattern with a residual small amount of SUMO1 and SUMO2/3 staining in a speckled pattern upon TAK-981 treatment (Fig. 6A, right). Using capillary-liquid chromatography-nanospray tandem mass spectrometry, proteins that were significantly enriched in SUMO immunoprecipitations relative to IgG control (1.3 fold enrichement, p = 0.05) were first identified and the set of these proteins with loss of enrichment by TAK-981 treatment was then determined (see Additional file 1: Methods for additional details). We found a total of 153 proteins enriched within the SUMO immunoprecipitation fractions (n = 45 SUMO1, n = 108 SUMO2/3) (Fig. 6B, left). DAVID functional enrichment analysis shown SUMO1 and SUMO2/3 fractions to be separately enriched in proteins with distinct functions, with a heavy predominance of RNA processing, splicing, and ribosomal biogenesis proteins within the SUMO1 fraction while the SUMO2/3 fraction was much more diversified, containing a variety of transcription factors, chromatin modulatory proteins, and RNA binding proteins (Fig. 6C, right and Additional file 1: Fig. S10). As expected, multiple known sumoylation targets from previous studies were identified, including Topoisomerase I (TopI), Top2A, kinesin family member 4A (KIF4A), Myc associated factor X (MAX), the dual specificity Max associated transcription factor (MGA), PML, TRIM24, SAFB, and MIF, in addition to several others that have not been previously described, including PKC-beta, PLC-gamma2, and multiple RNA splicing enzymes. Interaction mapping with STRING shown clustering of proteins within distinct functional interacting groups (Fig. 6C), with the free SUMO proteins located at a central location interfacing with signaling, mitosis/DNA damage, transcription, and RNA splicing. TAK-981 was effective at reducing 82% of the enriched SUMO associated proteins, with an overall deeper reduction of SUMO2 associated proteins compared to SUMO1, with several showing a near complete elimination from the SUMO immunoprecipitations (Fig. 6D). Interestingly, there was an overall differential reduction of proteins within specific functional groups, with proteins involved in transcription (MAX, MGA, ARID4A/B, and PML) showing the greatest reduction, DNA processing and signaling (TopI, Top2A/B, PKC-beta) with moderate reduction, and proteins involved in RNA splicing (SNRPG, SART1, U2AF2) with the least reduction (Fig. 6D, right). These results indicate that MCL cells have a diverse sumoylation program with a variety of functions capable of being targeted, albeit differentially, by TAK-981.

### TopIIA is an important sumoylation target in mitotic MCL cells leading to loss of centromere localization

**Fig. 7:**
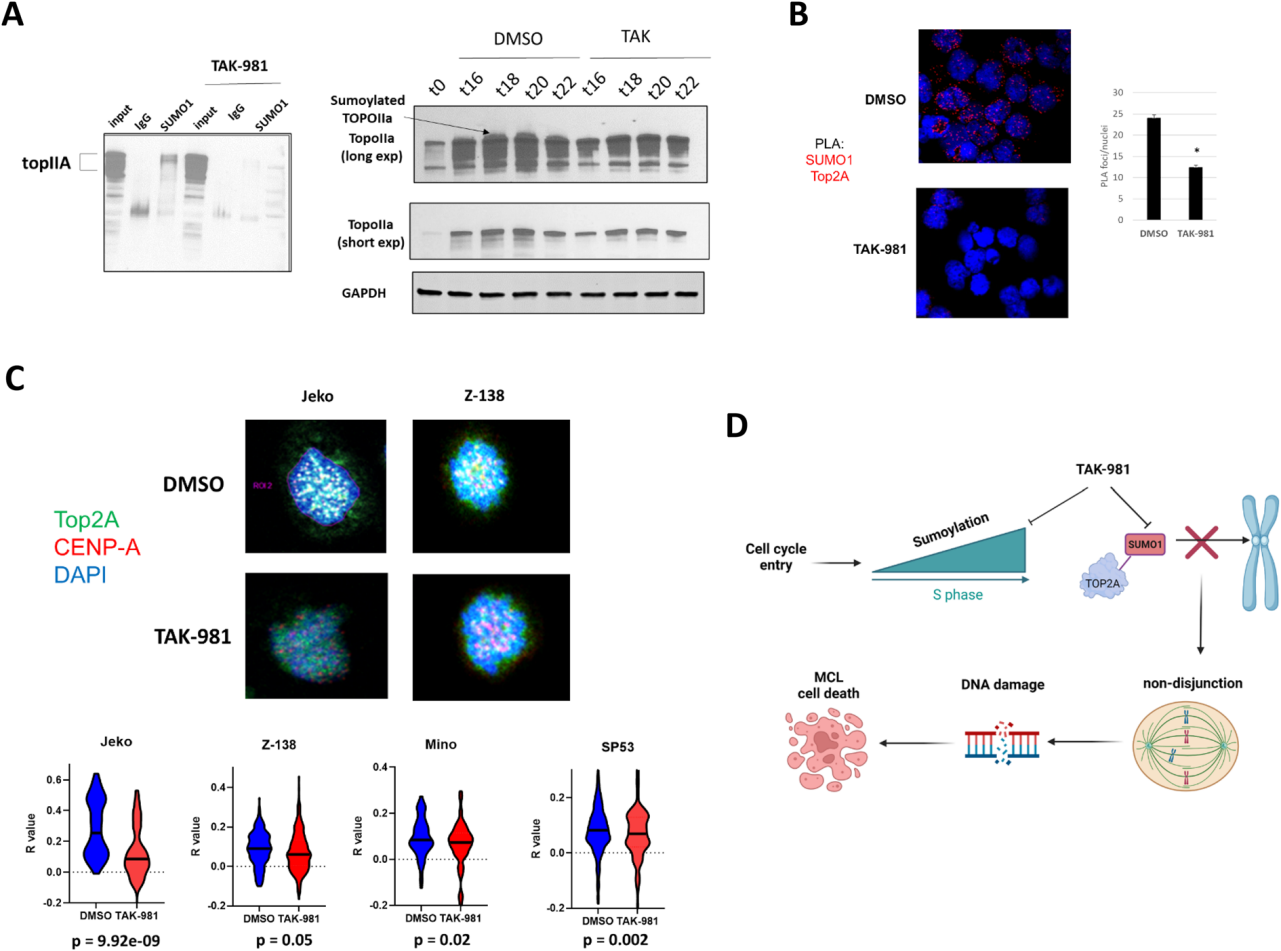
Inhibition of sumoylation with TAK-981 results in loss of centromeric localization of TopIIA in MCL cells **A**, left. Jeko cell were synchronized with Palbociclib (500 nM, 24 h) followed by drug washout and treatment with nocodazole (50 ng/mL) either in the presence of DMSO or TAK-981 (100 nM) for 24 h. Lysates were immunoprecipitated with antibodies directed towards SUMO1 and blotted for TopIIA. **A**, right. Jeko cells were synchronized with palbociclib (500 nM) and treated with either DMSO or TAK-981 (100 nM). Lysates were prepared at the indicated time points after washout and blotted for TopIIA. **B**, Jeko cells prepared as above were washed out of drug and fixed after 15 min. PLA for SUMO1 and topoIIA was performed. Number of individual PLA signals was quantified (n = 90 cells per condition). **C** Jeko (n = 88), Z-138 (n = 270), Mino (n = 80) and SP53 (n = 214) cells were prepared as above and microscopy was performed for TopoIIA and CENP-A to mark centromeric regions. The extent of colocalization of TopoIIA and CENP-A in either DMSO or TAK-981 treated cells was determined by cellsense (see “Methods” section n = 2 independent experiments per cell line). **D** Diagram of mechanism of desumoylation mediated cell death in MCL cells. Created on Biorender.com

Of the sumoylated protein targets discovered in MCL cells, we focused our attention on Top2A which was previously shown to be part of the proliferative gene expression-based model with prognostic significance in MCL [24]. Top2A undergoes localization to centromeric regions during late S phase and into mitosis where it serves a crucial chromosome decatenation function required for proper chromosome segregation, a process thought to be mediated by SUMO1 conjugation [30]. Top2A has also recently been discovered to be crucial for maintaining the structure of mitotic chromosomes [31]. We have shown that Top2A expression strongly correlated with that of SAE1 and SAE2 (Fig. 3A), overall suggesting that Top2A and the sumoylation pathway may be cooperating to maintaining mitotic fidelity in proliferative MCL. We first validated the results from our proteomic experiment by immunoblot and found Top2A to be enriched within the SUMO1 fraction and readily lost with TAK-981 treatment (Fig. 7A, left). Consistent with this, we found a higher molecular weight form of Top2A that appeared strongest upon late S phase/G2 entry which was readily lost in TAK-981 treated cells (Fig. 7A, right). We further confirmed the association of SUMO1 and Top2A in Jeko cells using PLA (Fig. 7B). As expected, TAK-981 treatment resulted in a significant loss of PLA signals consistent with our immunoprecipitation results (Fig. 7B). We next evaluated the localization of Top2A at centromeres by colocalization with the centromeric histone core subunit, CENP-A. (Fig. 7C). We confirmed that loss of sumoylation did not affect either the deposition or localization of CENP-A to centromeric regions (Additional file 1: Fig. S11). We found a significant decrease in the centromeric localization of Topo2A with TAK-981 treatment in Jeko (p < 0.001), Mino (p = 0.02), and SP53 cells (p = 0.002) (Fig. 7C). A smaller decrease was present in Z-138 cells which did not reach statistical significance (p = 0.05), suggesting there may be other mechanisms contributing to the mitotic dysregulation seen in these cells. However, it is also possible that we did not capture when Top2A was maximally localized to centromeres given their more rapid exit into mitosis seen in these cells following nocodazole washout (Additional file 1: Fig. S9). Taken together, our results suggest MCL cells have a complex, multi-functional sumoylation program required for survival with mitotic regulation through Top2A localization being an important function of the sumoylation pathway in mitotically active MCL cells (Fig. 7D).

## Discussion

In this study, we show that MCL cells have upregulation of the sumoylation pathway, more specifically at the level of the E1 enzymes SAE1/SAE2 (Fig. 1B, C). Sumoylation upregulation is not only prognostically significant among MCL patients but is also required for the proliferation and survival of MCL cells (Figs. 1D, E, 2A). The high correlation of expression of cell cycle, chromosome segregation and centromere related genes with SAE1 and SAE2 upregulation in MCL patient samples (Fig. 3A) along with the significant degree of mitotic dysregulation seen in many MCL cell lines and primary MCL samples after inhibition of SAE2 (Fig. 3B, C) strongly suggests that upregulation of the sumoylation pathway is serving a crucial role in maintaining mitotic fidelity in MCL cells in the face of their high degree of proliferation and severe cell cycle checkpoint dysfunction. In this regard, it is interesting to note that MCL samples with mutations in either p53 or ATM tended to have the highest degree of polyploidy with loss of sumoylation (Fig. 3B, C).

One of the possible roles of sumoylation enzyme upregulation is to maintain the dynamic changes in sumoylated proteins upon transition through different phases of the cell cycle (Fig. 4A), such as the case for directing Top2A to centromeres for decatenation of DNA. The fraction of sumoylated proteins present prior to mitosis entry in MCL cells contains several functional groups, including proteins involved in mitosis and signaling, transcription factors and chromatin remodeling proteins, and RNA splicing proteins, many of which were lost with TAK-981 treatment. Although the number of proteins directly involved in the mechanics of mitosis was unexpectedly small, it is possible that just a few, such as Top2A, KIF4A, and TopI may be playing a significant role in mediating the mitotic dysfunction in MCL. In this regard, loss of Top2A function at centromeres is consistent with the phenotype observed, with formation of chromatin bridges, non-disjunction, and DNA damage induced in mitotic cells. Mouse models of SUMO E3 ligase Ran-BP2 haploinsufficiency showed similarities to TAK-981 treated MCL cells, with significant mitotic dysregulation, aneuploidy, and Top2A centromeric mislocalization in lymphoid cells [32]. Our cell cycle phase experiments with TAK-981 (Fig. 5) further suggest that this centromeric activity is likely completed prior to entry into mitosis, which may account for the lack of effect of TAK-981 if not given prior to mitosis entry. Finally, the cooperativity between the sumoylation enzymes SAE1 and SAE2 and Top2A in MCL cells is also suggested by the high correlation of the expression levels of these enzymes in MCL cells (Fig. 3A).

One of the most interesting results of this study is the differential nature of sumoylated protein targeting by TAK-981, with transcription factors and chromatin remodeling enzyme sumoylation being the most readily lost with TAK-981 treatment. Sumoylated transcriptional factors targeted by TAK-981 may be more relevant in cases where inhibition of sumoylation can lead to cell death without requiring cells to be in the cell cycle, as in the case of CCMCL1 and MCL patient samples 4 and 5 (Fig. 3B, C). This may be the case in other lymphomas such as T-cell lymphomas where survival may be dependent on sumoylated oncogenic transcription factors such as the Histone Deactylase (HDACs) or the signal transducer and activator of transcription (STAT) family of proteins (reviewed in [33]). Overall, our results suggest mechanisms of action of sumoylation inhibition in MCL cells which will require further characterization using specific cell lines or primary MCL samples that carry these specific phenotypes.

Our findings of the direct cytotoxic mechanism of action of TAK-981 compliments a recent study showing a potent indirect anti-lymphoma response mediated by activation of a type I interferon response leading to increases in intratumoral T cells and NK cells [17]. Several studies have shown that chromosomal instability can also induce immune clearance by multiple mechanisms, including increased surface protein expression resulting in enhanced NK cell recognition [34], activation of cGAS-STING signaling through cytosolic immunostimulatory DNA formed from lagging chromosomes [35], and the increased presence of calreticulin on the cell surface resulting in enhanced immune recognition [36]. It is exciting to speculate that the inherent chromosomal instability in MCL cells imposed by loss of sumoylation may cooperate with the direct innate immune activating activity of desumoylation by priming polyploid MCL cells for rapid immune clearance, but further studies in immunocompetent mouse models of MCL will be needed to establish this connection.

There are some limitations to our study. Direct pulldown of endogenous sumoylated proteins in cells that do not express mutated versions of SUMO proteins likely reduces our sensitivity for detecting sumoylated proteins, especially for low affinity interactions. Also, as we used cells synchronized at a specific point of the cell cycle, we may have missed relevant proteins transiently sumoylated to carry out their function prior to when we performed our immunoprecipitation experiments. However, this is in lieu of likely increasing the specificity for more relevant sumoylated proteins carrying out specific functions during this point of the cell cycle, which is likely the case for Top2A.

The studies conducted here have uncovered potent direct cytotoxicity of targeting sumoylation in MCL and laid the groundwork for further mechanistic studies. Additional studies with TAK-981 in combination with other targeted therapies such as ibrutinib, venetoclax, and lenalidomide, will hopefully identify how to best apply desumoylation therapy in MCL and lead to improved patient outcomes, particularly in patients with highly proliferative MCL.

## Supplementary Information

**Additional file 1: Fig. S1**. (Left) B-cell were isolated from the peripheral blood from healthy donors and cultured in the absence (left) or presence (right) of cytokines and a CD40L expressing fibroblasts as previously described((1)). Flow cytometry was performed for the indicated markers. (Top right) Resting and activated B cells were fixed and stained with PI for cell cycle analysis. (Bottom right) Lysates were collected from resting and activated B-cells and blotted for p-Btk, total Btk, c-myc, and GAPDH. **Fig. S2**. (top) UMAP plots from 4 different patients with leukemic MCL showing lineage specific clustering of cells. (bottom) Dot plots verifying lineage specific marker enrichment among the individual clusters. **Fig. S3**. UMAP plots compiled from 4 different patients with leukemic MCL (left) or a reference PMBC B-cell data set (right) showing their distinct transcriptional states with an overall small amount of normal B-cells in leukemic MCL samples. **Fig. S4**. Jeko cells were retrovirally transduced with lentiviruses encoding either a non-targeting sh or an sh directed against SAE1. Cells were GFP sorted and the total viable cells in each was enumerated 3 days after sorting (n=3 biological replicates). **Fig. S5**. B cells and MCL cell lines were treated with TAK-981 (100 nM, 24 hours). Lysates were prepared and blotted for an antibody recognizing the SUMO-TAK-981 adduct (above) and GAPDH (below). **Fig. S6**. Representative cell cycle profiles of primary MCL patients samples taken at the time of beginning TAK-981 treatment in Fig.3B. **Fig. S7**. Jeko cells were synchronized with palbociclib (500 nM) and treated with either DMSO or TAK-981 (100nM) and lysates. Cell were fixed at the indicated time points and cell cycle distribution was determined of PI stained cells. **Fig. S8**. Z-138 cells were synchronized with palbociclib (500 nM) and treated with either DMSO or TAK-981 (100nM) and lysates. Cell were fixed at the indicated time points and cell cycle distribution was determined of PI stained cells. **Fig. S9**. (Top) Z-138 cells were synchronized with Palbociclib (500 nM, 24 hours) followed by drug washout and treatment with nocodazole (50 ng/mL) either in the presence of DMSO or TAK-981 (100 nM) for 24 hours. Both drugs were then washed out and cells were collected every hour for cell cycle analysis and lysates were prepared for protein for SUMOylation levels. (Bottom) Z-138 cells were synchronized with Palbociclib (500 nM, 24 hours) followed by drug washout and treatment with nocodazole (50 ng/mL) for 24 hours. Cells were then treated with either DMSO or TAK-981 for 3 hours. Cells were then washed from drugs and treated with either DMSO or TAK-981 and collected every hour for cell cycle analysis and lysates were prepared for protein for SUMOylation levels. **Fig. S10**. Results of DAVID functional annotation analysis performed on proteins enriched for sumoylation for SUMO1 (top) and SUMO2/3 (bottom). The percentage of SUMOylated proteins within individual functional annotations are shown along with the false discovery rate (FDR). **Fig. S11.** (Top). Jeko cells were synchronized with Palbociclib (500 nM, 24 hours) followed by drug washout and treatment with nocodazole (50 ng/mL) either in the presence of DMSO or TAK-981 (100 nM) for 24 hours. Both drugs were then washed out and cells were fixed and stained for CENP-A. Representative confocal images of cells or chromosomes are shown. (Bottom) Enumeration of CENP-A foci from DMSO or TAK-981 treated cells was performed using cellSens software. Results are representative of two independent experiments.

## Data Availability

For scRNA seq datasets, processed data with installation instructions are available as an R data package at https://datadryad.org/stash/share/tK1XG9bdwk8dZD1_-AZXIvz1fHfLURZU15dXhSCNsm0. Analysis code is publicly available at https://github.com/blaserlab/hanel_lapo. For all other original data, please contact Dr. Lapo Alinari (Lapo.Alinari@osumc.edu).

## Abbreviations

AO: Acridine Orange
ARID: A-T Rich Interaction Domain
ASPM: Assembly factor for spindle microtubules
ATM: Ataxia Telangiectasia mutated
BAFF: B-cell activating factor
BTK: Bruton’s Tyrosine Kinase
CAR: Chimeric Antigen Receptor
CDC: Cell division control
CDK1: Cyclin dependent kinase 1
CENP-A: Centromere protein A
CGAS: Cyclic GMP-AMP synthase
DAVID: Database for Annotation, Visualization, and Integrated Discovery
ERC: End removal criteria
FSC: Forward Scatter
GFP: Green fluorescent protein
H2B: Histone 2B
HRP: Horseradish peroxidase
IGF1: Insulin-like growth factor 1
IL: Interleukin
KIF4A: Kinesin Family Member 4A
MAX: Myc associated factor X
MGA: MAX gene associated protein
MCL: Mantle Cell Lymphoma
MIF: Macrophage Migration Inhibitory Factor
MIR: Micro RNA
MS: Mass-spectrometry
Myc: Myelocytomatosis protein
NHL: Non-hodgkin’s lymphoma
NK: Natural killer
OS: Overall Survival
PBMC: Peripheral blood mononuclear cell
PDX: Patient derived xenograft
PIAS: Protein inhibitor of activated STAT
PLA: Proximity ligation Assay
PML: Promyelocytic leukemia
PI: Propidium Iodide
PKC: Protein Kinase C
PLC: Phospholipase C
RANBP2: Ran-binding protein 2
RARA: Retinoic acid receptor alpha
RIPA: Radioimmunoprecipitation assay
R/R: Relapsed/Refractory
SAE: SUMO activating enzyme
SAFB: Scaffold attachment factor B
SART1: U4/U6.U5 tri-SnRNP-associated protein 1
scRNA: Single cell RNA
SEM: Standard error of the mean
SENP: Sentrin specific protease
shRNA: Short hairpin RNA
SNRPG: Small Nuclear-Ribonucleoprotein Polypeptide G
SSC: Side Scatter
STING: Stimulator of Interferon Genes
STRING: Search tool for the Retrieval of Interacting Genes/Proteins
SUMO: Small Ubiquitin-Like Modifier
TAK: Takeda
TOP1: Topoisomerase I
TOP2A: Topoisomerase 2 A
TP53: Tumor Protein 53
TRIM24: Tripartite Motif containing 24
U2AF2: U2 auxiliary factor 2
UBA2: Ubiquitin like Modifier activating enzyme 2
UBC9: Ubiquitin conjugating enzyme 9
UBE2I: Ubiquitin conjugating enzyme E2I
UMAP: Uniform Manifold Approximation and Projection
