# Supplementary material for: A sumoylation program is essential for maintaining the mitotic fidelity in proliferating mantle cell lymphoma cells
Source: Exp Hematol Oncol. 2022 Jul 13;11:40. doi: 10.1186/s40164-022-00293-y (PMC9277803; doi:10.1186/s40164-022-00293-y)
Supplement: Supplementary file 1 — Additional file 1: Fig. S1. (Left) B-cell were isolated from the peripheral blood from healthy donors and cultured in the absence (left) or presence (right) of cytokines and a CD40L expressing fibroblasts as previously described((1)). Flow cytometry was performed for the indicated markers. (Top right) Resting and activated B cells were fixed and stained with PI for cell cycle analysis. (Bottom right) Lysates were collected from resting and activated B-cells and blotted for p-Btk, total Btk, c-myc, and GAPDH. Fig. S2. (top) UMAP plots from 4 different patients with leukemic MCL showing lineage specific clustering of cells. (bottom) Dot plots verifying lineage specific marker enrichment among the individual clusters. Fig. S3. UMAP plots compiled from 4 different patients with leukemic MCL (left) or a reference PMBC B-cell data set (right) showing their distinct transcriptional states with an overall small amount of normal B-cells in leukemic MCL samples. Fig. S4. Jeko cells were retrovirally transduced with lentiviruses encoding either a non-targeting sh or an sh directed against SAE1. Cells were GFP sorted and the total viable cells in each was enumerated 3 days after sorting (n=3 biological replicates). Fig. S5. B cells and MCL cell lines were treated with TAK-981 (100 nM, 24 hours). Lysates were prepared and blotted for an antibody recognizing the SUMO-TAK-981 adduct (above) and GAPDH (below). Fig. S6. Representative cell cycle profiles of primary MCL patients samples taken at the time of beginning TAK-981 treatment in Fig.3B. Fig. S7. Jeko cells were synchronized with palbociclib (500 nM) and treated with either DMSO or TAK-981 (100nM) and lysates. Cell were fixed at the indicated time points and cell cycle distribution was determined of PI stained cells. Fig. S8. Z-138 cells were synchronized with palbociclib (500 nM) and treated with either DMSO or TAK-981 (100nM) and lysates. Cell were fixed at the indicated time points and cell cycle distribution was determine [file 40164_2022_293_MOESM1_ESM.docx]

**Supp. Fig.1**


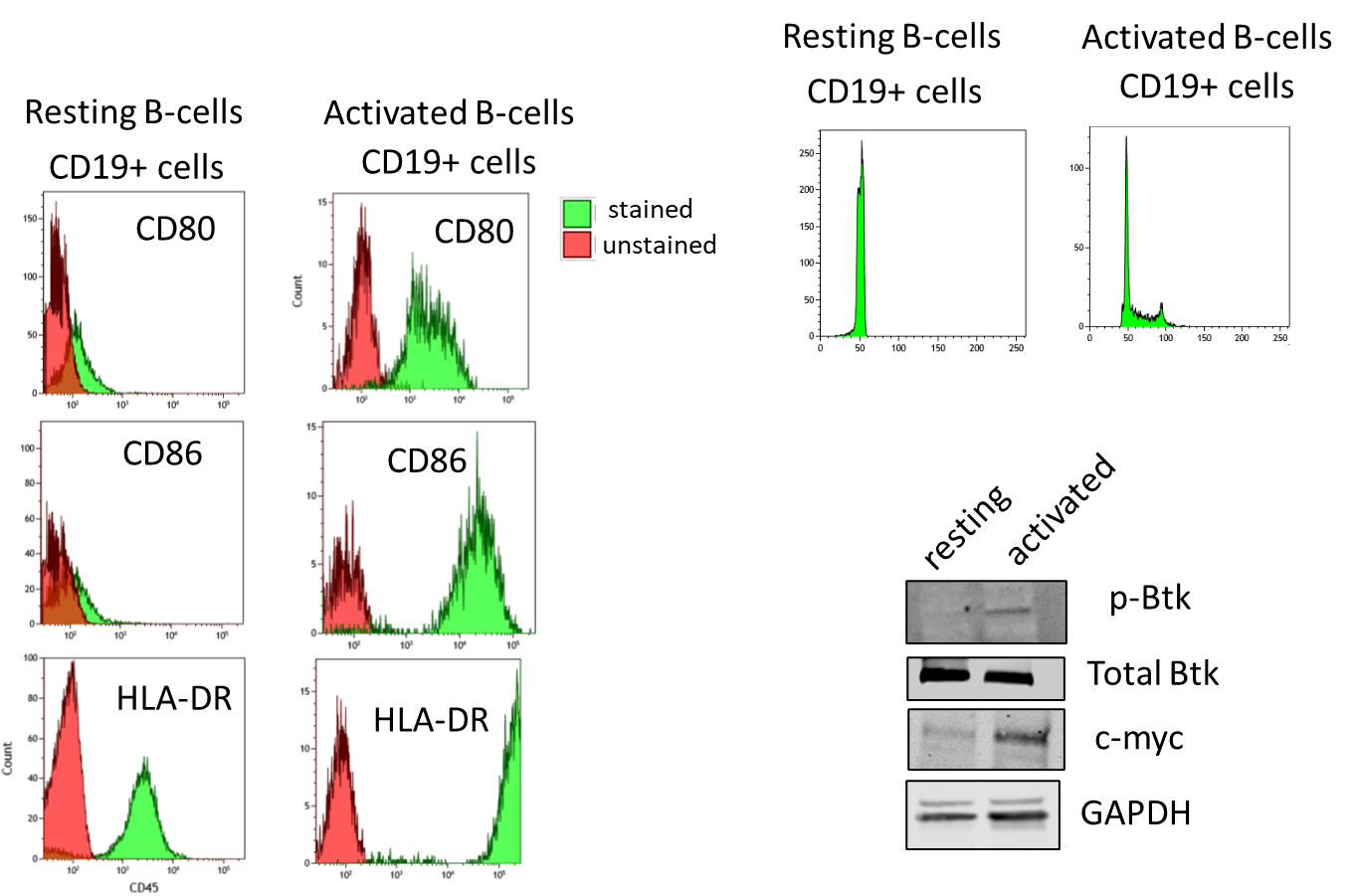


**Supp. Fig1. (Left)** B-cell were isolated from the peripheral blood from healthy donors and cultured in the absence (left) or presence (right) of cytokines and a CD40L expressing fibroblasts as previously described((1)). Flow cytometry was performed for the indicated markers. (**Top right**) Resting and activated B cells were fixed and stained with PI for cell cycle analysis. (**Bottom right**) Lysates were collected from resting and activated B-cells and blotted for p-Btk, total Btk, c-myc, and GAPDH.

**Supp. Fig.2**


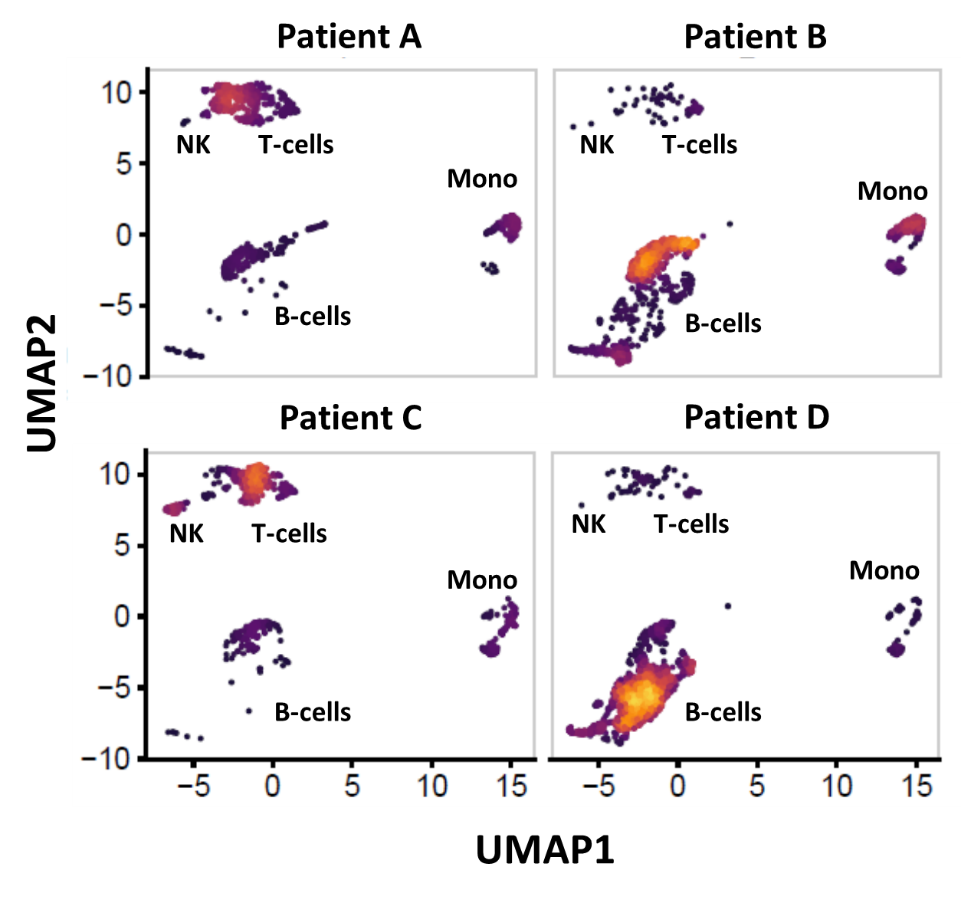


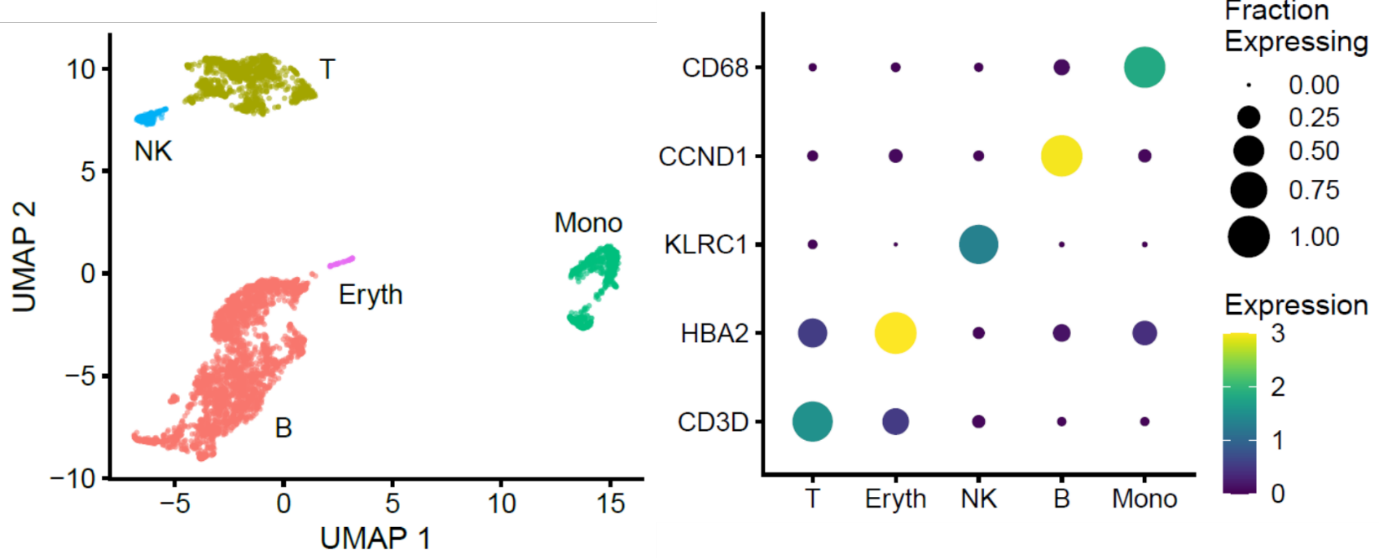


**Supp. Fig.2. (top)** UMAP plots from 4 different patients with leukemic MCL showing lineage specific clustering of cells. (**bottom**) Dot plots verifying lineage specific marker enrichment among the individual clusters.

**Supp. Fig.3**


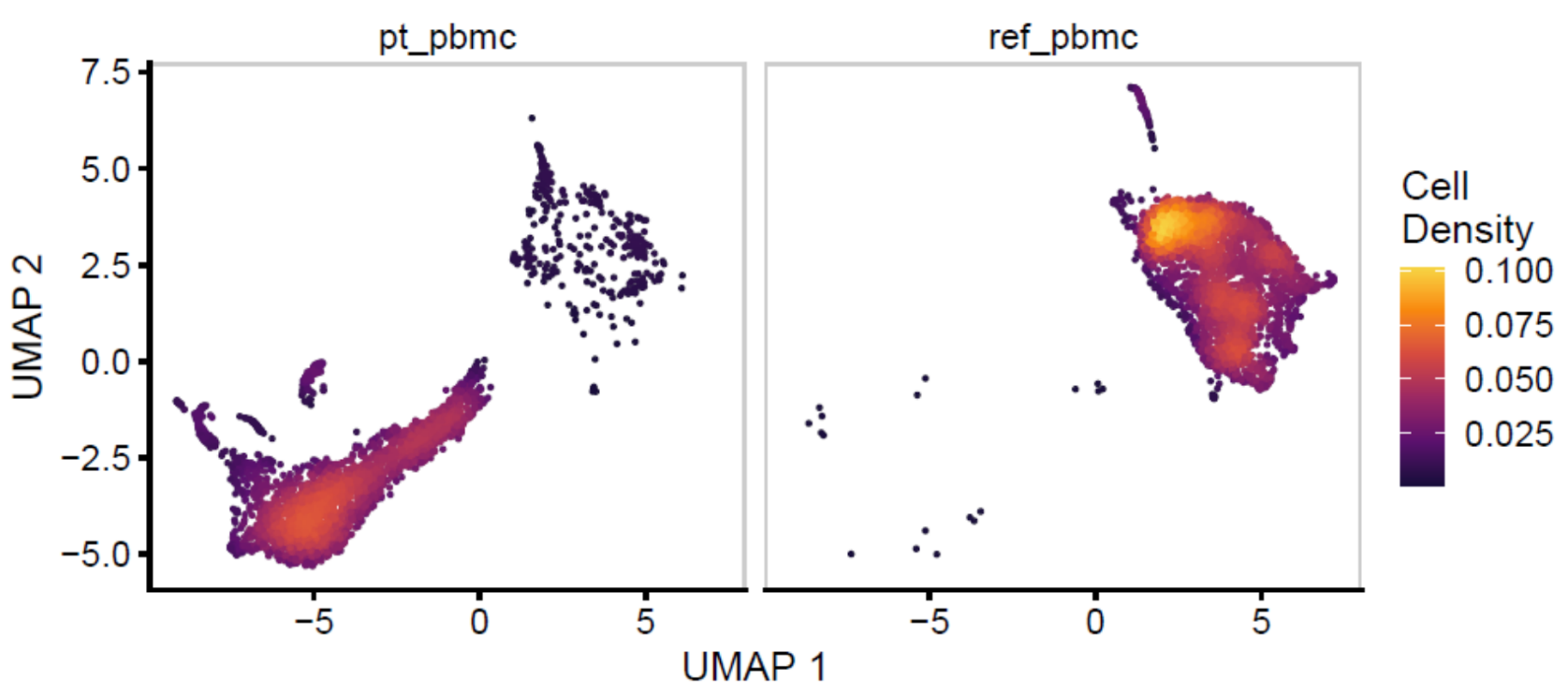


**Supp. Fig.3.** UMAP plots compiled from 4 different patients with leukemic MCL (**left**) or a reference PMBC B-cell data set (**right**) showing their distinct transcriptional states with an overall small amount of normal B-cells in leukemic MCL samples.

**Supp. Fig.4**


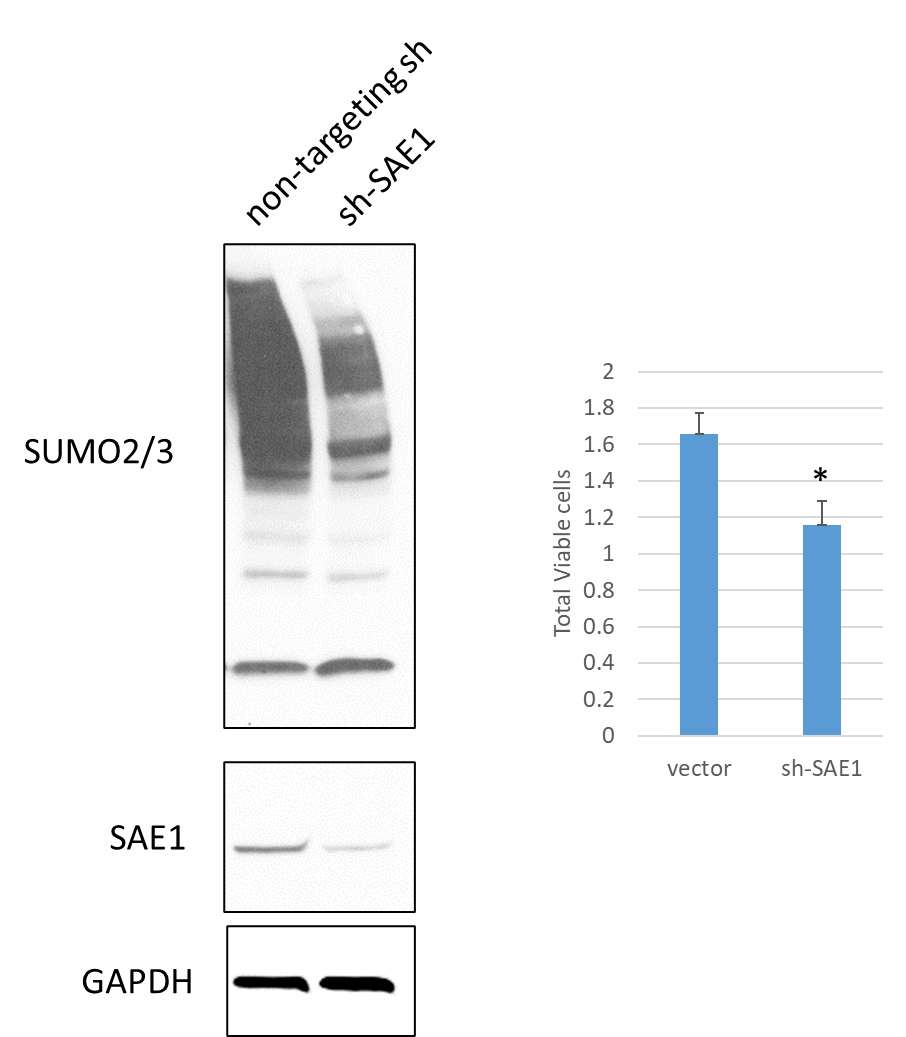


**Supp. Fig.4.** Jeko cells were retrovirally transduced with lentiviruses encoding either a non-targeting sh or an sh directed against SAE1. Cells were GFP sorted and the total viable cells in each was enumerated 3 days after sorting (n=3 biological replicates).

**Supp. Fig.5**


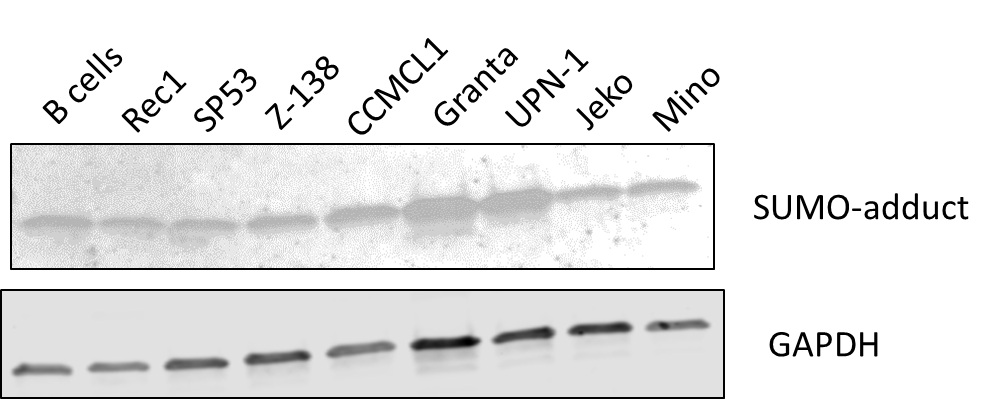


**Supp. Fig.5.** B cells and MCL cell lines were treated with TAK-981 (100 nM, 24 hours). Lysates were prepared and blotted for an antibody recognizing the SUMO-TAK-981 adduct (above) and GAPDH (below).

**Supp. Fig.6**


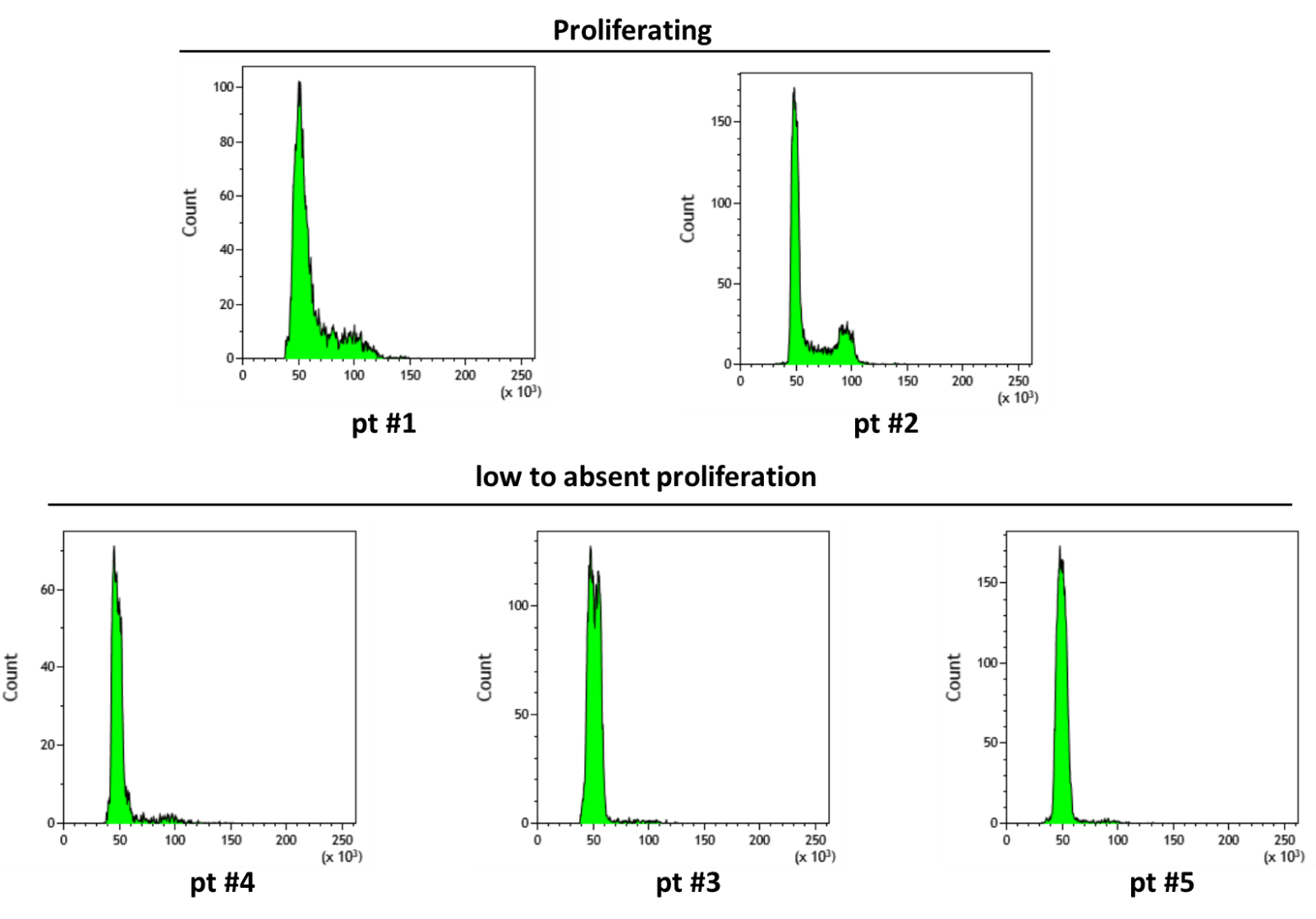


**Supp. Fig.6.** Representative cell cycle profiles of primary MCL patients samples taken at the time of beginning TAK-981 treatment in **Fig.3B**.

**Supp. Fig.7**


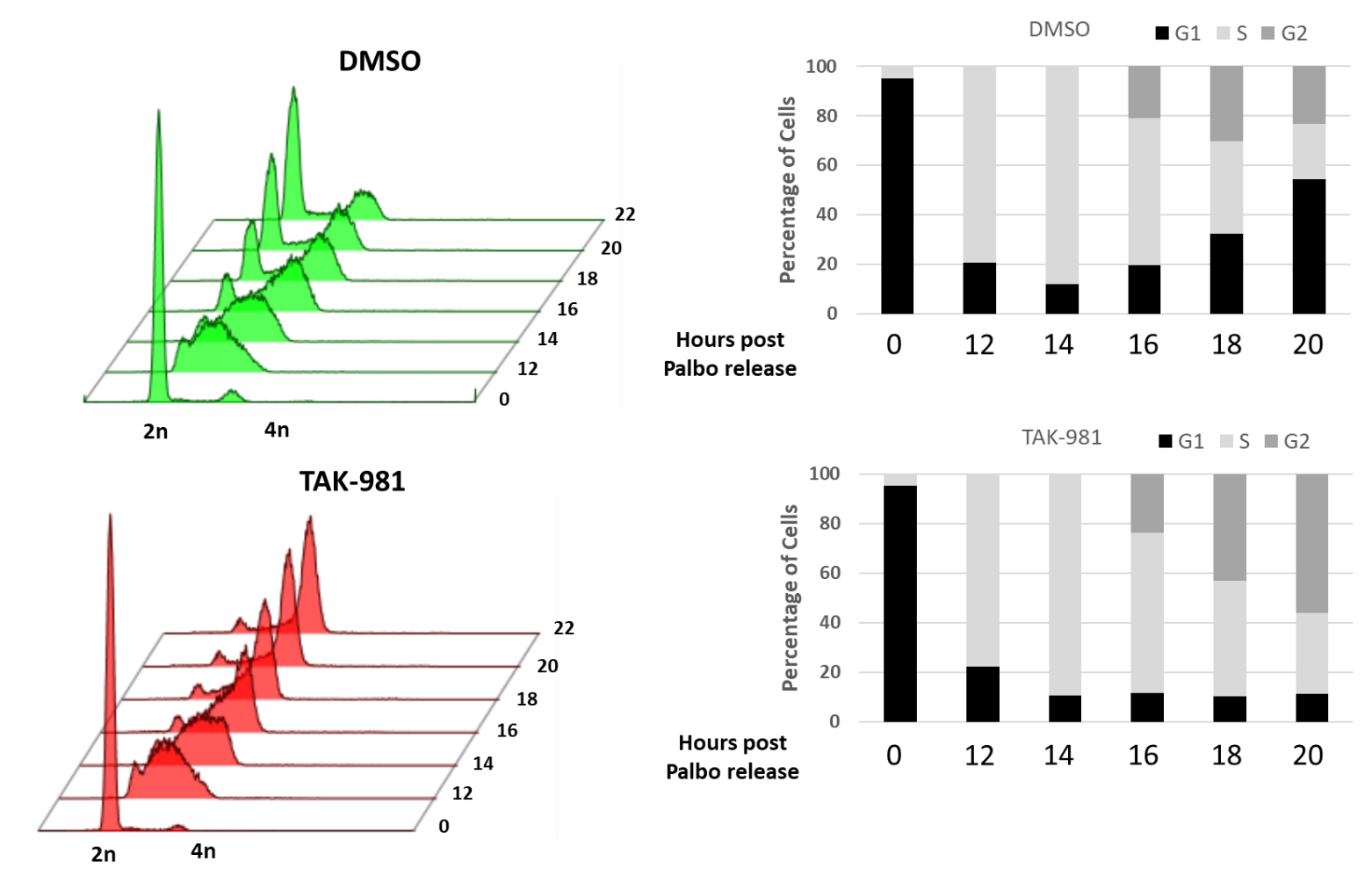


**Supp. Fig.7.** Jeko cells were synchronized with palbociclib (500 nM) and treated with either DMSO or TAK-981 (100nM) and lysates. Cell were fixed at the indicated time points and cell cycle distribution was determined of PI stained cells.

**Supp. Fig.8**


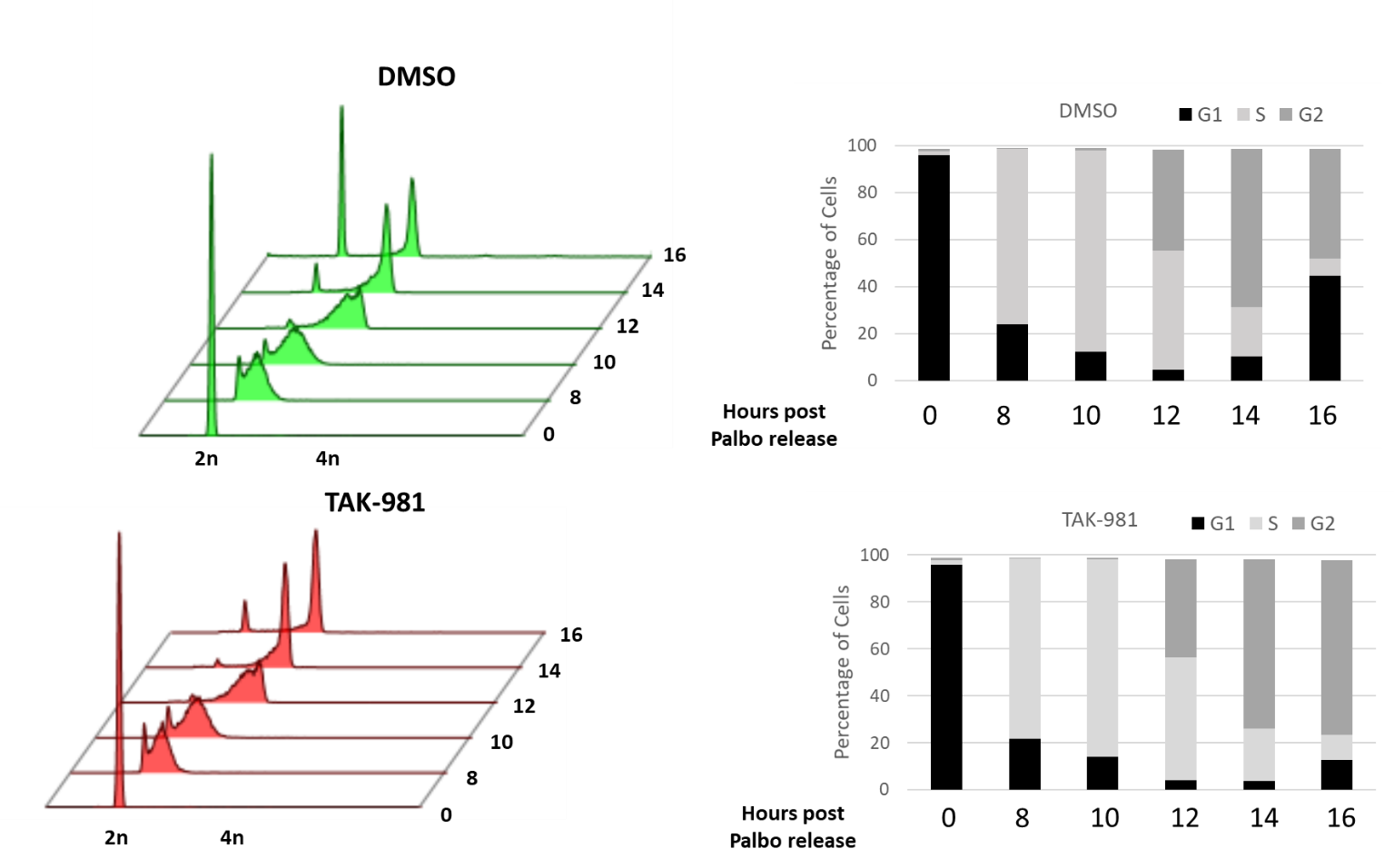


**Supp. Fig.8.** Z-138 cells were synchronized with palbociclib (500 nM) and treated with either DMSO or TAK-981 (100nM) and lysates. Cell were fixed at the indicated time points and cell cycle distribution was determined of PI stained cells.

**Supp. Fig.9**


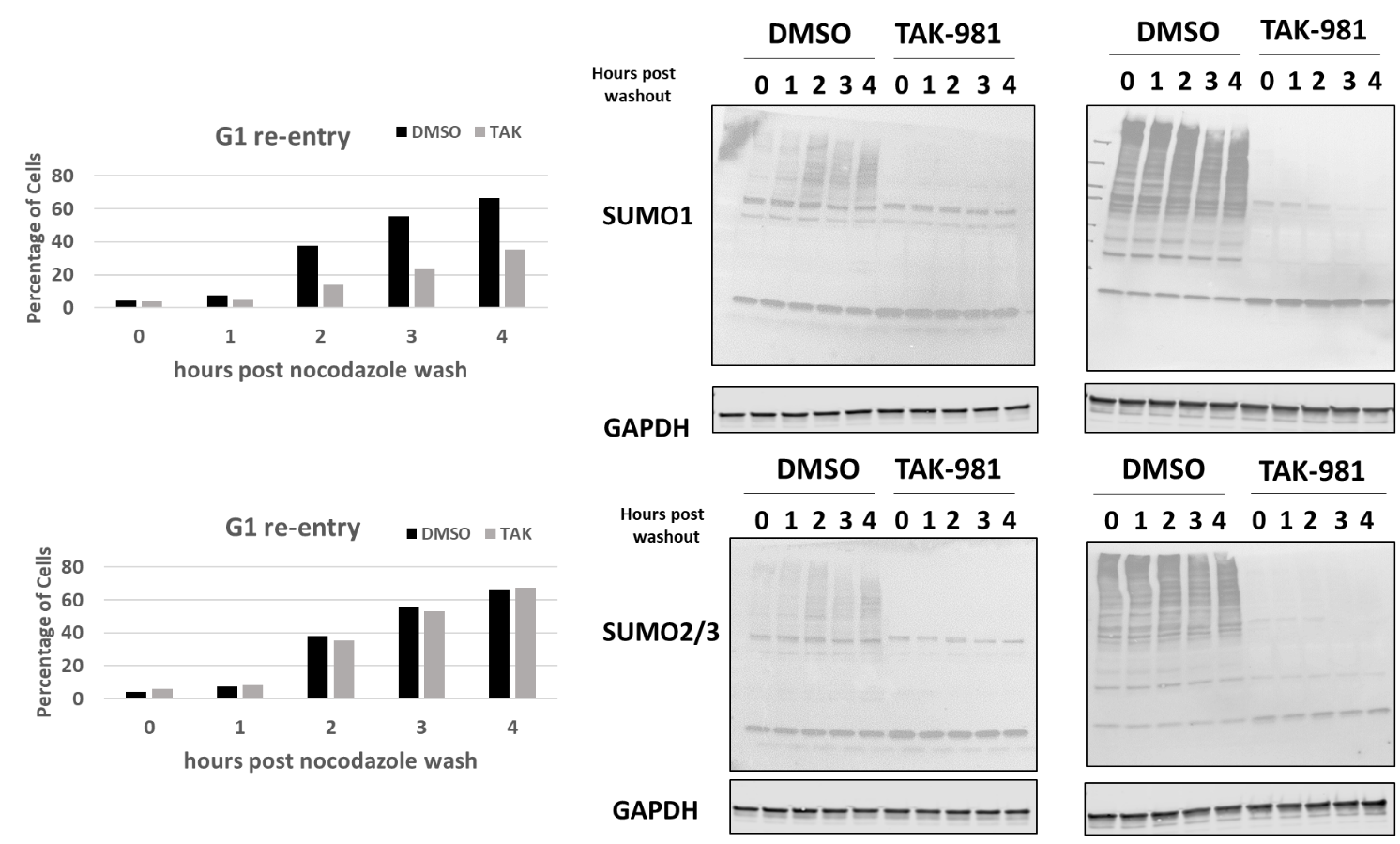


**Supp. Fig.9. (Top)** Z-138 cells were synchronized with Palbociclib (500 nM, 24 hours) followed by drug washout and treatment with nocodazole (50 ng/mL) either in the presence of DMSO or TAK-981 (100 nM) for 24 hours. Both drugs were then washed out and cells were collected every hour for cell cycle analysis and lysates were prepared for protein for SUMOylation levels. **(Bottom)** Z-138 cells were synchronized with Palbociclib (500 nM, 24 hours) followed by drug washout and treatment with nocodazole (50 ng/mL) for 24 hours. Cells were then treated with either DMSO or TAK-981 for 3 hours. Cells were then washed from drugs and treated with either DMSO or TAK-981 and collected every hour for cell cycle analysis and lysates were prepared for protein for SUMOylation levels.

**Supp. Fig.10**


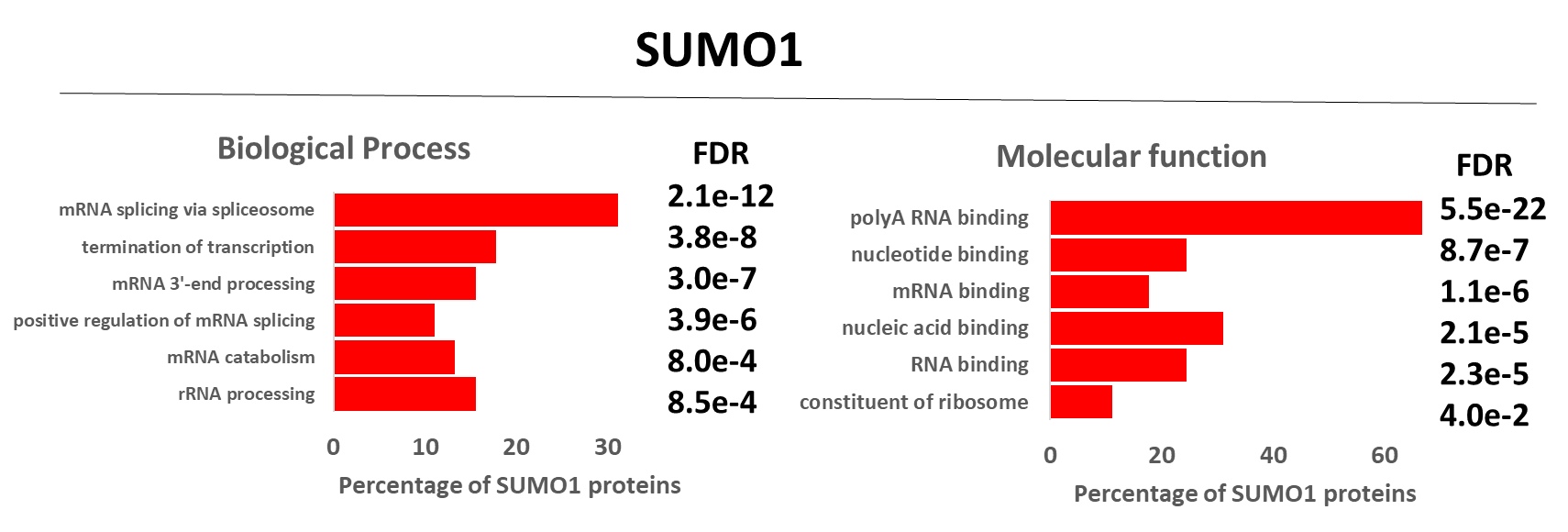


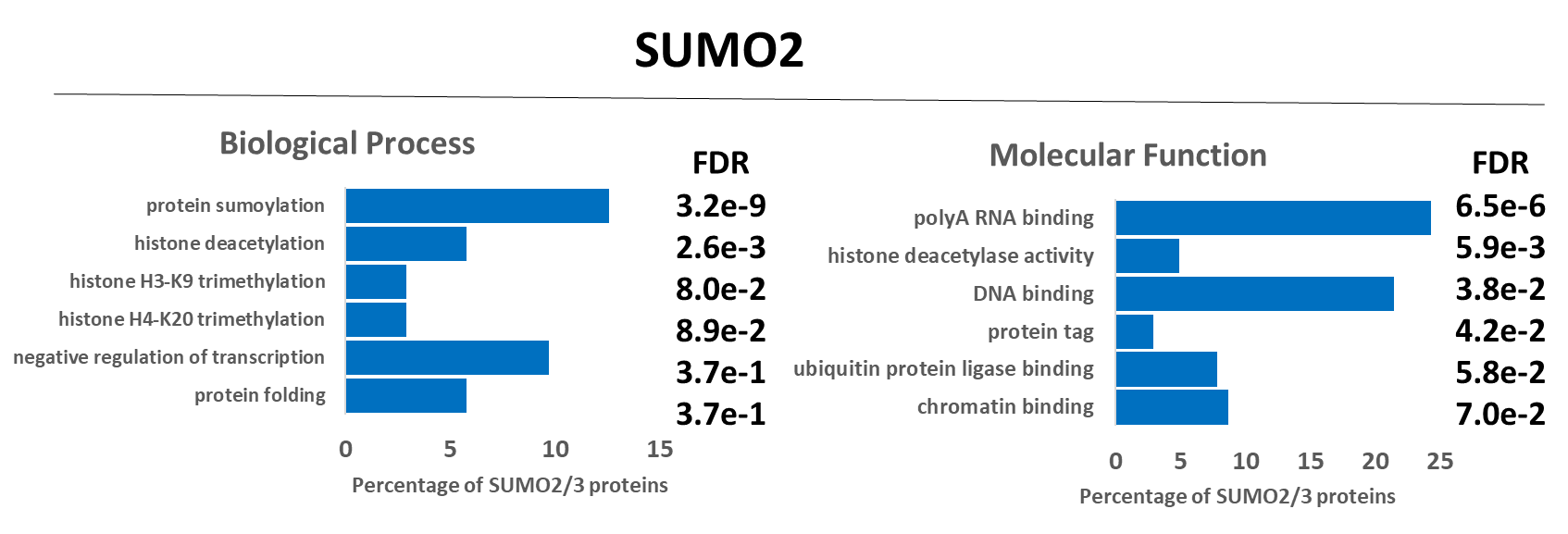


**Supp. Fig.10.** Results of DAVID functional annotation analysis performed on proteins enriched for sumoylation for SUMO1 (top) and SUMO2/3 (bottom). The percentage of SUMOylated proteins within individual functional annotations are shown along with the false discovery rate (FDR).

**Supp. Fig.11**


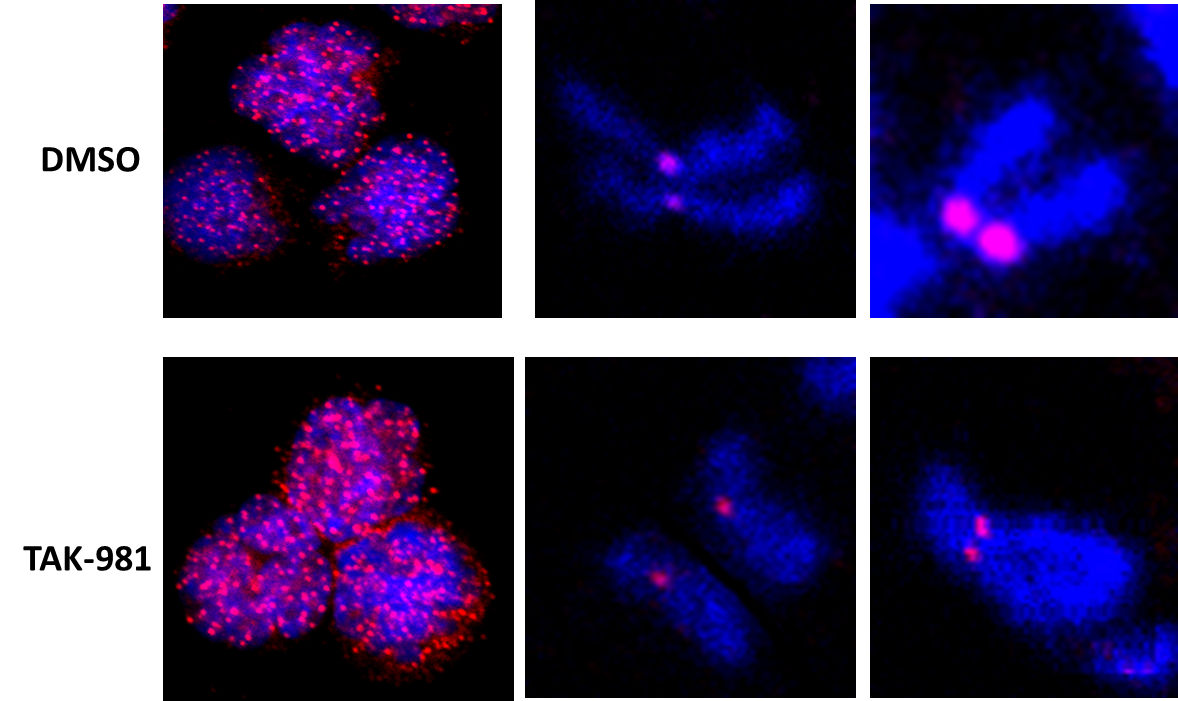


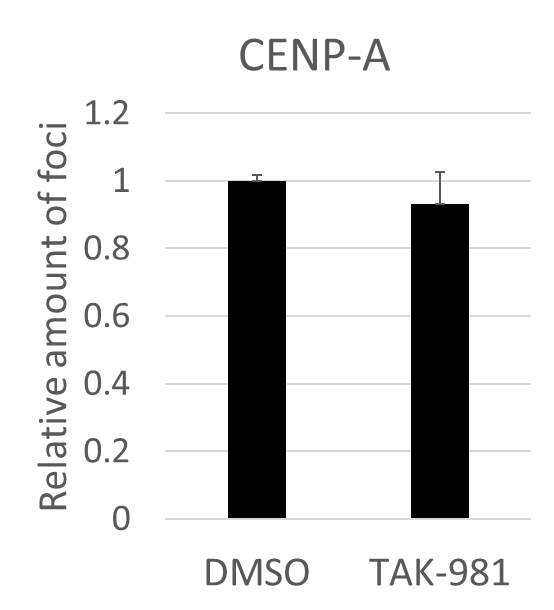


**Supp. Fig.11 (Top).** Jeko cells were synchronized with Palbociclib (500 nM, 24 hours) followed by drug washout and treatment with nocodazole (50 ng/mL) either in the presence of DMSO or TAK-981 (100 nM) for 24 hours. Both drugs were then washed out and cells were fixed and stained for CENP-A. Representative confocal images of cells or chromosomes are shown. **(Bottom)** Enumeration of CENP-A foci from DMSO or TAK-981 treated cells was performed using cellSens software. Results are representative of two independent experiments

**Supplementary Methods**

**scRNA sequencing**

*Single cell capture, library generation and sequencing*

Rehydrated, washed cells were stained with ethidium homodimer (Live/Dead Viability/Cytotoxicity Kit, Invitrogen L3224) and counted using a Keyence BZX-700 digital fluorescence microscope and ImageJ software. Single cells were captured using the 10X Genomics Single Cell 3’ v3 kit targeting recovery of 2000 cells per sample according to the manufacturer’s instructions. Illumina-compatible sequencing libraries were generated from barcoded, amplified cDNA and sequenced on an Illumina HiSeq 4000 instrument (Nationwide Children’s Hospital Institute for Genomic Medicine). More than 69,000 reads per cell were obtained.

*Data processing and analysis*

FASTQs, BAM files, and cell barcode-gene matrices were generated using Cellranger v3.1.0 using the 10X-supplied GRCh38 reference. QC functions from *scater* were used to remove cells with a proportion of counts mapped to mitochondrial genes that was more than 2 median absolute deviations (MADs) above the median or a feature count that was more than 2 MADs below the median(2). High likelihood doublets were predicted using *Doubletfinder* and removed. Patient to patient variation was reduced by aligning according to mutual nearest neighbors with *Batchelor.* Dimension reduction, clustering, top marker identification, gene module analysis and quantification of gene expression was performed using custom functions derived from Monocle3. Cell clustering was performed using the partitioning method. Descriptive labels were attached to cell partitions by inspection of top specific markers (**supp. fig1**) and by label transfer from reference data sets using *Seurat*. Pseudobulk expression profiles from 2966 MCL patient B cells and an equal number of B cells sampled from 8 patients in the normal reference data set were generated using Monocle3 functions. Differential expression testing between patient and reference pseudobulk profiles was performed using DESeq2 with each donor representing a biological replicate.

*Data and code availability*

Raw data files are available in GEO with accession number XXX. Processed data with installation instructions are available as an R data package at <https://datadryad.org/stash/share/tK1XG9bdwk8dZD1_-AZXIvz1fHfLURZU15dXhSCNsm0>.

Analysis code is publicly available at <https://github.com/blaserlab/hanel_lapo>.

**Live cell microscopy**

Jeko and Z-138 cells were transduced with a H2B-GFP lentivirus (Origene) and placed under puromycin selection (1ug/mL). GFP expression was confirmed by flow cytometry. Cells were taken off puromycin selection for at least 72 hours prior to downstream experiments. Cell lines were treated with palbociclib (500 nM) for 24 hours followed by washout and release into either DMSO or TAK (100 nM) and plated in 96 well plates. Cells were imaged using the Incucyte Zoom System (Essen BioScience). Cells were imaged every 5 minutes from 6 hours to 48 hours after the start of image collection at 20X magnification. Individual cells were tracked from the beginning to the end of mitosis based on previously reported morphological criteria(3).

**Confocal microscopy**

Cells were fixed with 4% paraformaldehyde for 10 minutes at 37°C. Fixed cells were then washed three times with PBS (pH 7.4) followed by permeabilization with 0.1% Triton X-100 for 15 minutes at room temperature. Cells were then washed with PBS (pH 7.4) three times, blocked with 2% BSA for 2 hrs at 37°C, and then incubated overnight at 4°C with the indicated primary antibody either singly or with a cocktail of two for colocalization assay. On day 2, cells were washed three times with PBS (pH 7.4) and incubated for 2 hrs at 37°C with secondary antibodies fluorescently labelled with alexa fluor-488 and alexa fluor-647. The cells were cytospun on the glass slides after three PBS (pH 7.4) washes. Nuclei were stained with 4′, 6-diamidino-2-phenylindole (DAPI) containing mount medium (abcam, ab104139) for 5 minutes at room temperature and then coverslipped for confocal microscopy observation. Fluorescently labelled cells were analyzed by Olympus Filter FV1000 Laser Scanning Confocal Imaging Systems and the data was analyzed by Cellsens microscopy image analysis software. For every experiment, at least 100 cells were counted in control and each treatment group from each independent run with at least two independent runs performed for each experiment.

**Proximity Ligation Assay (PLA)**

The TAK-981 treated Jeko cells were fixed with 4% paraformaldehyde for 10 minutes at 37°C. They were permeabilized with 0.1% Triton-X-100 for 15 minutes at room temperature, washed in PBS and blocked in Duolink Blocking buffer for 1 hr at 37°C. Primary antibodies were diluted (1:100) in Duolink Antibody Diluent and incubated overnight at 4 °C. Where appropriate, cells were counterstained using Anti-IgG (CST#3900S and CST#5415S), rabbit anti-SUMO1 (abcam; ab32058), mouse anti-TOP2A (R&D systems; MAB6540), and rabbit anti-TOP2A (Cell Signaling Technology: 12286S) primary antibodies overnight. The cells were washed three times for 5 min with Duolink in situ wash buffer A (Sigma; DUO82049), followed by incubation with Duolink in situ PLA^®^ Probe anti-rabbit MINUS (Sigma; DUO92006) and anti-mouse PLUS (Sigma; DUO92001) for 2 hrs at 37°C, ligation for 1.5hrs at 37°C and overnight amplification at 37°C. Ligation and amplification steps of the PLA were performed using the Duolink in situ Detection Reagents red kit (Sigma; DUO92008) according to the manufacturer’s instructions. Following the PLA, Nuclei were stained with 4′,6-diamidino-2- phenylindole (DAPI) containing mount medium (abcam, ab104139) for 5 minutes at room temperature and then cover-slipped for confocal microscopy observation. PLA signal was analyzed by using Olympus Filter FV1000 Laser Scanning Confocal Imaging Systems. PLA spots were counted in cell lines using IMARIS software. PLA scores were determined by normalizing the number of PLA spots counted in each sample to the number of cell counted in the same sample.

**Co-immunoprecipitation**

For SUMO1 immunoprecipitation, anti-SUMO1 antibodies were covalently coupled to the M270 epoxy Dynabeads according to the manufacturer's protocol (Invitrogen). For SUMO2/3 interaction, agarose conjugated SUMO2/3 affinity beads (Cytoskeleton, Inc.). Whole cell extracts were incubated with these coupled antibodies for 30-35 minutes at 4°C. Beads were collected, washed three times and processed as per kit protocol and bound proteins were analyzed by western blotting. Beads were washed with 50 mM ammonium bicarbonate (50uL each time depends on beads volume) three times. After the third wash, 5uL of DTT (5ug/uL in50 mM ammonium bicarbonate) was added and the sample incubated at 56C for 15 min. After the incubation, 5uL of ammonium bicarbonate with iodoacetamide added (15 mg/ml in 50mM) and the sample kept in dark at room temperature for 30 min. 500ng of sequencing grade-modified trypsin (Promega, Madison WI) prepared in 50 mM ammonium bicarbonate was added to the sample reaction and carried out at 37C overnight. Additional 50mM ammonium bicarbonate was added to make the final volume of the samples to 100uL. The reaction was quenched the next morning by adding acetic acid for acidification. Supernatant were removed and concentrated for LC/MSMS analysis.

**Mass Spectrometry Analysis**

Capillary-liquid chromatography-nanospray tandem mass spectrometry (Capillary-LC/MS/MS) of protein identification was performed on a Thermo Scientific orbitrap Fusion mass spectrometer equipped with an [nanospray FAIMS Pro™ Sources](https://www.thermofisher.com/order/catalog/product/ES081)  operated in positive ion mode.  Samples (4.0 µL) were separated on an easy spray nano column (Pepmap^TM^ RSLC, C18 3µ 100A, 75µm X150mm Thermo Scientific) using a 2D RSLC HPLC system from Thermo Scientific. Each sample was injected into the µ-Precolumn Cartridge (Thermo Scientific) and desalted with 0.1% Formic Acid in water for 5 minutes. The injector port was then switched to inject and the peptides were eluted off of the trap onto the column. Mobile phase A was 0.1% Formic Acid in water and acetonitrile (with 0.1% formic acid) was used as mobile phase B. Flow rate was set at 300nL/min. Mobile phase B was increased from 2% to 16% in 105 min and then increased from 16-25% in 10 min and again from 25-85% in 1 min and then kept at 95% for another 4 min before being brought back quickly to 2% in 1 min. The column was equilibrated at 2% of mobile phase B (or 98% A) for 15 min before the next sample injection.

MS/MS data was acquired with a spray voltage of 1.95 KV and a capillary temperature of 305 °C is used. The scan sequence of the mass spectrometer was based on the preview mode data dependent TopSpeed™ method: the analysis was programmed for a full scan recorded between *m/z* 375-1500 and a MS/MS scan to generate product ion spectra to determine amino acid sequence in consecutive scans starting from the most abundant peaks in the spectrum in the next 3 seconds. To achieve high mass accuracy MS determination, the full scan was performed at FT mode and the resolution was set at 120,000 with internal mass calibration. Three FAIMS compensation voltage (cv=-50, -65 and -80v) were used for data acquisition. The AGC Target ion number for FT full scan was set at 4 x 10^5^ ions, maximum ion injection time was set at 50 ms and micro scan number was set at 1. MSn was performed using HCD in ion trap mode to ensure the highest signal intensity of MSn spectra. The HCD collision energy was set at 32%. The AGC Target ion number for ion trap MSn scan was set at 3.0E4 ions, maximum ion injection time was set at 35 ms and micro scan number was set at 1. Dynamic exclusion is enabled with a repeat count of 1 within 60s and a low mass width and high mass width of 10ppm.

Data were searched using Mascot Daemon by Matrix Science version 2.7.0 (Boston, MA) via ProteomeDiscoverer (version 2.4 Thermo Scientific,) and the database searched against the most recent Uniprot databases. The mass accuracy of the precursor ions were set to 10ppm, accidental pick of 1 ^13^C peaks was also included into the search. The fragment mass tolerance was set to 0.5 Da. Carbamidomethylation (Cys) is used as a fixed modification and considered variable modifications were oxidation (Met) and deamidation (N and Q). Four missed cleavages for the enzyme were permitted. A decoy database was also searched to determine the false discovery rate (FDR) and peptides were filtered according at 1% FDR. Proteins identified with at least two unique peptides were considered as reliable identification. Any modified peptides are manually checked for validation. For each protein identified, significance was determined by comparing spectral counts between SUMO IPs and IgG with a significance level of p=0.05. Under the assumption of a linear relationship between spectral counts a protein abundance(4), percent inhibition was determined by dividing the average spectral count in TAK-981 treated SUMO IPs by DMSO treated SUMO Ips.

**Methods References**

1. Su KY, Watanabe A, Yeh CH, Kelsoe G, Kuraoka M. Efficient Culture of Human Naive and Memory B Cells for Use as APCs. J Immunol. 2016;197(10):4163-76.

2. McCarthy DJ, Campbell KR, Lun AT, Wills QF. Scater: pre-processing, quality control, normalization and visualization of single-cell RNA-seq data in R. Bioinformatics. 2017;33(8):1179-86.

3. Sivakumar S, Daum JR, Gorbsky GJ. Live-cell fluorescence imaging for phenotypic analysis of mitosis. Methods Mol Biol. 2014;1170:549-62.

4. Liu H, Sadygov RG, Yates JR, 3rd. A model for random sampling and estimation of relative protein abundance in shotgun proteomics. Anal Chem. 2004;76(14):4193-201.

**Catalog of Key Antibodies Used in this Study**

| **WB/IP/IF/PLA antibodies** | **Source** |
| --- | --- |
| SUMO1 | Abcam (ab32058) WB, IP, IF, PLA |
| SUMO2/3 | Cytoskeleton (ASM23) IP, abcam (ab81371) WB |
| SUMO-adduct | Takeda Development Center Americas, Inc (MIL 113-67-2) |
| TOP2A | CST (12286S) WB; R&D systems (MAB6540), Abcam (ab52934) IF, PLA |
| CENP-A | Abcam (ab13939) WB; Invitrogen #MA1-20832 IF |
| SAE1/UBLE1A | CST #13585S WB |
| SAE2/UBA2 | CST #5293S WB |
| UBC9 | CST #4786S WB |
| GAPDH | Invitrogen #MA5-15738, mouse WB; CST #2118S, rabbit, WB |
